# Stillbirth and newborn data quality and use and related input and process factors: findings of the IMPULSE study in Ethiopia

**DOI:** 10.7189/jogh.16.04231

**Published:** 2026-07-17

**Authors:** Dawit Fisseha, Firehiwot Abathun, Lorenzo Giovanni Cora, Bogale Worku, Belete Belgu, Aderajew Mekonnen Girmay, Tiyese Chimuna, Meles Solomon, Hailu Abebe, Ilaria Mariani, Mary Ayele, Muhumuza Kananura Rornald, Jacqueline Minja, Ousman Mouhamadou, Francesca Tognon, Joy E Lawn, Giovanni Putoto, Donat Shamba, Peter Waiswa, Tamrat Awell, Marzia Lazzerini

**Affiliations:** 1 Doctors with Africa CUAMM, Addis Ababa, Ethiopia; 2 WHO Collaborating Centre for Maternal and Child Health, Institute for Maternal and Child Health, IRCCS Burlo Garofolo, Trieste, Italy; 3 Ethiopian Pediatrics Society, Addis Ababa, Ethiopia; 4 Ethiopian Midwives Association, Addis Ababa, Ethiopia; 5 Ethiopian Public Health Institute, Addis Ababa, Ethiopia; 6 UNICEF Ethiopia, Addis Ababa, Ethiopia; 7 Maternal Child and Adolescent Health Service Lead Executive Office, Ministry of Health Ethiopia, Addis Ababa, Ethiopia; 8 Makerere University of Public Health, Makerere, Uganda; 9 African Population and Health Research Centre, Nairobi, Kenya; 10 Ifakara Health Institute in Tanzania, Ifakara, Tanzania; 11 Doctors with Africa CUAMM, Bangui, Central African Republic; 12 Doctors with Africa CUAMM, Padua, Italy; 13 London School of Hygiene & Tropical Medicine, London, UK; 14 Ministry of Health Ethiopia: Strategic Affairs Lead Executive Office, Addis Ababa, Ethiopia

**Keywords:** newborn, stillbirth, data quality and use, Ethiopia, PRISM

## Abstract

**Background:**

High quality data is mandatory when shaping action-oriented policies aimed at reducing preventable neonatal deaths. Ethiopia has a high a neonatal mortality rate, but there has been limited assessment of the quality and use of newborn data, and its contributing factors.

**Methods:**

We conducted a cross-sectional study in Ethiopia from November 2022 to September 2023 in 35 sites: 24 facilities, 10 subnational data offices, and the Ministry of Health. We collected data using Every Newborn – Measurement Improvement for Newborn & Stillbirth Indicators tools and analysed them per the Performance of Routine Information System Management User’s Kit.

**Results:**

The major strengths identified in our analysis included governance at subnational data offices (80–90%), use of quality improvement standards (100%), supervision quality (100%) in data offices, sites with internet access (92–100%), routine health information system (RHIS) designated staff (100%), data management in data offices (62–100%), data availability and completeness (91–98%) for denominator elements (total births, live births), and use of data to produce reports in data offices (100%). Regarding key gaps, data accuracy (registers *vs*. District Health Information Software 2) was low on all 10 newborn indicators (11–67%), while data use for performance review was suboptimal (≤90%) at both in facilities (12–66%) and data offices (21–90%). Key weaknesses in input and process factors included only 40% of data offices having a long-term financial plan for supporting RHIS, 8% of data offices and 16% of facilities having the minimum item bundle in working conditions, few sites having supplies of recording/reporting tools (15–68%, depending on the item), and supervision quality (58–62%), staff skills to perform RHIS tasks (16–40%), and data management (46–75%) being particularly low in facilities. An average of 57% of end users reported a need for improvement in the quality and use of newborn data, as well as in the studied enabling factors.

**Conclusions:**

Our findings highlight the specific strengths and weaknesses that need to be addressed to improve data quality and use in the assessed sample in Ethiopia.

Neonatal mortality poses a significant burden on global health, particularly in low- and middle-income countries (LMICs). Neonatal deaths do not only have an emotional and psychological effects on families but can also have broader societal implications, straining healthcare systems, impeding economic development, and exacerbating inequalities. There were an estimated 2.3 million neonatal deaths globally in 2023, with sub-Saharan Africa recording the highest neonatal mortality rate at 26 deaths per 1,000 live births [1]. Approximately 1.9 million stillbirths occur every year, nearly half of which (45%) take place in sub-Saharan Africa, even though African countries contribute to 30% of global live births [2,3]. Ethiopia specifically faces significant challenges in this context, with a neonatal mortality rate of 27.13 per 1,000 live births and a stillbirth rate of 20.56 per 1,000 total births [4,5]. The above rates suggest that Ethiopia is out of track for the global targets defined by the Sustainable Development Goals and emphasised by Every Newborn Action Plan (ENAP), aiming to reduce the neonatal mortality rate and stillbirth rate to 12 deaths per 1,000 live births by 2030 [2,5]. In fact, it is among the 10 countries with the highest burden of global maternal deaths, stillbirths, and newborn deaths in the world [6].

A robust health information system (RHIS) can facilitate accountability, inform quality improvement efforts, support informed management decisions, identify programme priorities, and monitor and evaluate progress toward national and subnational health targets [7,8]. Timely and accurate data are crucial in ending preventable stillbirth and neonatal mortality [6,9,10], with those produced by RHIS being especially critical [11]. Countries like Ethiopia, which are off track to meeting the global targets of newborn mortality and stillbirths, need timely and accurate data for planning, and for accelerating progress towards meeting global targets [9].

A systematic review highlighted that few studies documented stillbirth and newborn data quality in LMICs [12]. In Ethiopia, few previous investigations have focused on gaps in neonatal and stillbirth data quality and use, emphasising the need for more comprehensive and multi-setting research to identify underlying factors affecting this gap [13–16]. Systematic reviews of international literature, as well as studies conducted in Ethiopia, have shown that data quality and use is influenced by many underlying organisational, technical, and behavioural factors acting at different level of the health system (central, regional, district, facilities of different level), as well as the availability of resources in general [17–19].

We aimed to synthetically analyse the availability, quality, and utilisation of newborn and stillbirth indicators and data elements across selected regions and across different levels in the health system in Ethiopia (**Box 1**). By employing predefined, tested tools, specifically the Every Newborn – Measurement Improvement for Newborn & Stillbirth Indicators (EN-MINI) tools, an adaptation of the Performance of Routine Information System Management (PRISM) tools, we sought to evaluate these indicators rigorously across different sites in the country.

Box 1 **Key findings**

What was known before this study?

- High quality data and high data use are needed to prioritise and monitor interventions aimed at reducing preventable newborn mortality and morbidity.
- Previous assessments of data quality and use in Ethiopia did not focus on stillbirth and newborn indicators and/or were not comprehensively describing input and process factors.
- Decision-making and prioritisation can be challenging in the absence of a comprehensive evaluation and a synthetic summary of results.

What did we find and what does it mean?

- Despite several strengths, we observed gaps in data quality and use, with key being those related to data accuracy (registers *vs*. DHIS2) on all 10 newborn indicators assessed, and data use for performance reviews.
- We documented several specific gaps in inputs and process factors, particularly, but not uniquely, at facility level when compared to DHOs/MoH.

What is new about this study?

- We describe, for the first time and according a predefine PRISM framework and methodology, many underlying determinants of newborn and stillbirth data quality and use in Ethiopia, allowing for a better understanding of our findings.
- Most of the data were collected through direct observation, with a series of quality assurance procedures implemented.
- The synthetic reporting is a novelty aimed at offering a practical actionable high-level overview of findings, to favour interventions.

What is next for implementation?

- Our results can be used to prioritise actions and tailor local interventions to improve stillbirth and newborn data quality and use in Ethiopia during IMPULSE phase 2.
- Data can be further utilised to support the development of action plans for local implementation.
- The study can be easily replicated in other sites, and data can be further monitored to assess changes over time.

What research gaps remain?

- Further implementation research is needed to identify sustainable tailored interventions to improve stillbirth and newborn data quality and use in Ethiopia.
- The PRISM methodology could be further optimised by identifying summary indicators enabling assessing performance for each major domain, thus enabling multivariate analyses to further identify associations across indicators, and potential causal pathways.

## METHODS

**Figure 1:**
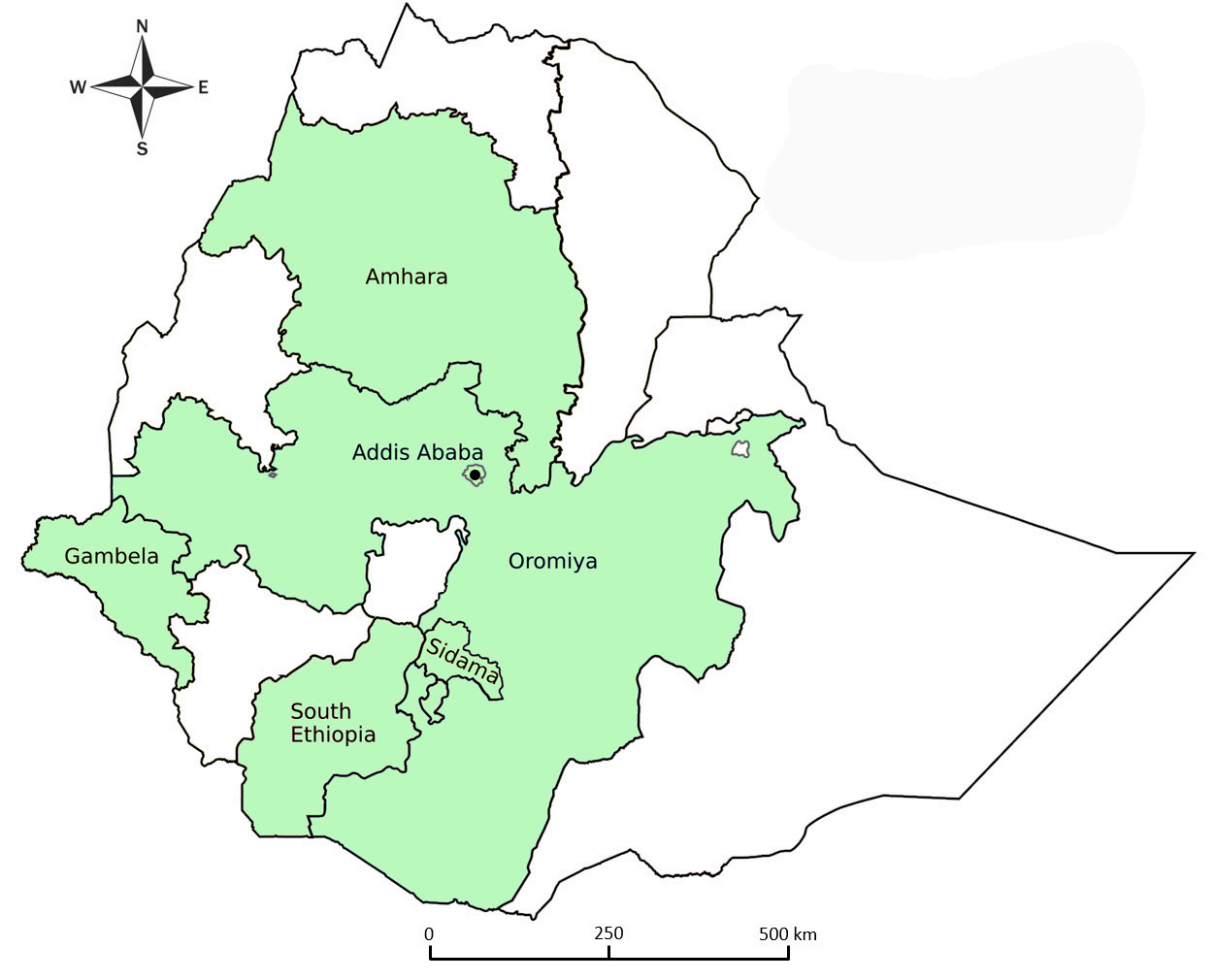
IMPULSE study map for the Ethiopian regions under assessment. Regions of Ethiopia assessed by the IMPULSE study are highlighted in green. The black dot represents the Addis Ababa city administration.

We conducted a cross-sectional study from November 2022 to September 2023 in five regions and one city administration of Ethiopia (**Figure 1**). We report our findings per the STROBE guidelines [20] (Table S1 in the **Online Supplementary Document**).

### Sampling and participants

According to the PRISM-adapted Lot Quality Assurance Sampling methodology, a minimum of 19 health facilities were to be assessed in each country within the IMProving qUaLity and uSE of newborn measures (IMPULSE) project. Regions were selected based on a balance of the following three criteria:

– heterogeneity, *i.e.* regions with different characteristics, including those underperforming in terms of maternal and neonatal mortality; hard-to-reach areas; or humanitarian settings;

– regions where the implementing agency, Doctors with Africa *Collegio Universitario Aspiranti Medici Missionari* (*CUAMM*) had an office/project that could facilitate coordination or other or other easy-to-reach regions (well-connected or near to the capital city);

– regions prioritised per request of the local ministries of health (MoHs).

**Figure 2:**
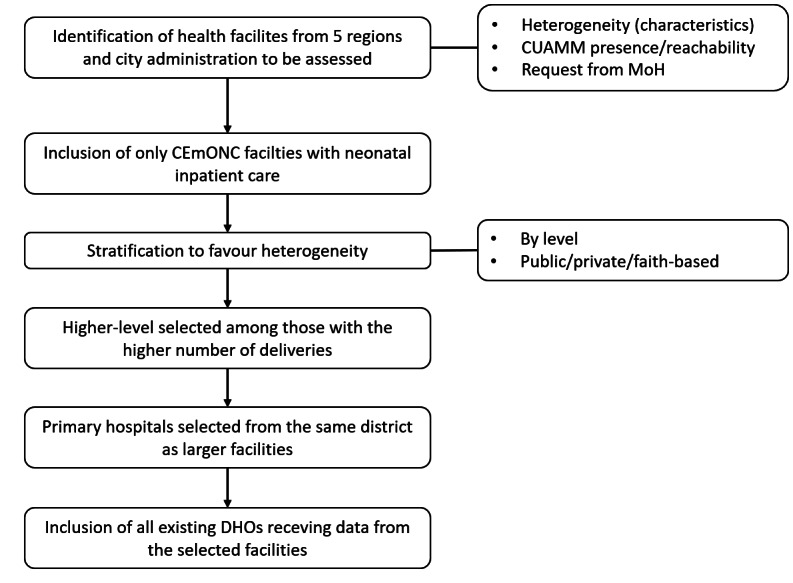
Flowchart for the sampling strategy of IMPULSE phase I. CEmONC – comprehensive emergency obstetric and newborn care, *CUAMM* – *Collegio Universitario Aspiranti Medici Missionari*, DHOs – district health offices, MoH – Ministry of Health.

We included only facilities providing comprehensive emergency obstetric emergency and newborn care with neonatal inpatient care. Within this criterion, we stratified the sample to include different levels and types of facilities (*i.e.* national- to primary-level hospitals; public and private hospitals, and faith-based care centers), whereby we predefined a fixed number of facilities in each category for each region in our sampling criteria (Table S2 in the **Online Supplementary Document**). We selected two second-level and one third-level referral public facilities in each region among those with the higher number of deliveries. For practicality, three public first-level hospitals (locally called ‘primary hospitals’) were selected per region in the same district as the larger facilities. We also included related health offices at district, regional, and central level, including all existing data offices receiving data from the selected facilities. We also assessed a national referral hospital (locally called ‘comprehensive specialised hospital’) in the capital city administration. Private and not-for-profit facilities were included if available in each region and allowed data collection (**Figure 2**). Due to the onset of conflicts in Ethiopia during the data collection period, the expected sample size had to be necessarily reduced in Amhara (five health facilities excluded) and Gambella (three health facilities excluded). Due to project resource constraints, only public hospitals were included in this region, while private facilities were included from the nearby region of Sidama (Table S3 in the **Online Supplementary Document**).

We enrolled respondents who were involved in data collection, compilation, and reporting in service delivery units, specifically from labour and delivery wards, neonatal wards, and postnatal ward, for end-user interviews. At district health offices (DHOs) and regional health bureaus, maternal and child health programme staff, and the policy planning, monitoring, and evaluation directorate participated in the data collection process. We also included health information technician officers at the facility level.

### Data collection

We collected data using standard operating procedures defined by the EN-MINI tools [21]. This set of open-access tools was collaboratively adapted from the PRISM tools, which have been used for over twenty years. The EN-MINI-PRISM tools were developed by the Every Newborn – Birth Indicators Research Tracking in Hospitals phase 2 research partners and further optimised and field tested (version 2) during the start-up phase of the IMPULSE study, in collaboration with the IMPULSE International Advisory Board and the four IMPULSE National Advisory Boards (one for each country) [21]. Most data were directly observed and/or extracted from existing relevant documents (*e.g.* guidelines, reports, meeting minutes). Data for assessment of data quality dimensions were collected from official facility registers of labour and delivery room, neonatal and postnatal wards. The EN-MINI toolkit includes ready-to-use digital data collection tools [21,22]. They were originally released in English and Swahili, and, before the start of data collection, were translated by study coordinators (FA, MA) into Amharic (for use in Ethiopia). EN-MINI tool 6, which included skill testing among the staff, was also translated into Afan Oromo.

The EN-MINI Tools [21] allow customisation of newborn indicators. For the IMPULSE study, we opted to assess availability of 16 indicators recommended by the ENAP/Every Woman Every Newborn Everywhere (EWEVEN) [23] and the data quality on 10 ENAP/EWENE key available and recommended indicators [24], including one maternal indicator as the tracer for neonatal and maternal measurements.

### Data quality assurance

Teams of three trained researchers performed the data collection under the supervision of experienced study coordinators (MA and FA), with both receiving training, including field practice and meetings to discuss and clarify doubts and questions. We also designed a file where all questions and answers were recorded and set up a WhatsApp group to solve any remaining issue in real-time. We followed predefined standard operating procedures for data collection: data were directly entered into SurveyCTO, an Open Data Kit-based secure-digital platform, and checks for data completeness and plausibility were performed periodically.

As further internal data quality check, during data collection, regular monitoring and evaluation of data collection were conducted by the study co-ordinators (MA and FA). Specifically, data were reviewed on a daily basis by an external supervisor (FT), using a pre-defined and tested Microsoft Excel file. Missing data or implausible data was discussed in real-time. Lastly, four rounds of interim analyses were also conducted by three independent data analysts (IM, PD, SG) to check data completeness, internal consistency, plausibility, in the first months of data collection. Results were discussed after each round, and data collection was optimised (e.g. missing data were collected) accordingly.

### Data analysis

We descriptively analysed the availability (presence or absence) of ENAP-recommended indicators in the national RHIS electronic system (the District Health Information Software 2 (DHIS2) in Ethiopia); 378 indicators included in the PRISM User’s Kit [25], summarised in an actionable synthetic figure, six additional questions collecting the users’ perspective regarding the need for improvements related to the RHIS for newborn and stillbirth data. The PRISM framework organises the indicators into technical, behavioural, and organisational categories (Figure S1 in the **Online Supplementary Document**), which we retained in our analysis. We further aggregated data in the synthetic figure at the level of subnational offices (district health offices (DHOs) and regional health bureaus, depending on availability) and facilities (first, second and third level of referral).

For the analysis of the 378 indicators included in the PRISM User’s Kit [25], we used its internal structure, categorisation, indicators, definitions, and wording. The PRISM User’s Kit and PRISM framework divide indicators in six major domains [21]:

– input factors: organisational (which further includes promotion of information culture and resources availability), technical, and behavioural factors;

– process factors: data management;

– output factors: data quality and data use.

We further organised the indicators within each of these six major domains in a variable number of subdomains based on the PRISM Users’ Kit [25]. To create the overall actionable synthesised figure of the selected indicators, we aggregated indicators in the same subcategory were by calculating an average percentage (as usually done in PRISM methodology [25]).

This analysis covered all indicators in the PRISM User’s Kit [25] collected as percentages or percentage scores, which we sought to make available for stakeholders a comprehensive assessment in a synthetic single page actionable format (Tables S6–12 in the **Online Supplementary Document**). The gap between perceived competence and observed skill was evaluated by calculating the difference between the related indicators, expressed as percentage points difference. We developed the indicator for key physical resources given the absence of a PRISM indicator for the physical resources, and was calculated as an average percentage, to align with the methodology proposed by the PRISM User’s Kit for all other indicators [25].

We assessed data quality for 10 recommended indicators: eight numerator data elements (stillbirths, institutional neonatal deaths, low birthweight, early initiation of breastfeeding, bag-mask-ventilation, kangaroo mother care, neonatal sepsis, and uterotonics to prevent post-partum haemorrhage) and two denominator data elements (total births and live births). Data accuracy among the two relevant key data sources (registers *vs*. DHIS2) in Ethiopia are entered in DHIS directly at facility level, and was calculated according to standard PRISM guidance on data reported over a three-month period (April to June 2022) as the percentage of sites with perfect accuracy (defined as perfect data matching, *i.e.* the ratio between the absolute number of records in registers vs those reported in DHIS2 equal to 1) and the number of sites with a data accuracy within ±10% (*i.e.* ratio between the absolute numbers reported in registers *vs*. those reported in DHIS2 ranging between 0.9 to 1.1). Only sites which delivered the service were included in the denominator, meaning no missing values could enter the calculations. We did not consider late reporting for this analysis, as it is described by the timeliness dimensionality.

We cross-checked the final results of the analyses against the results of the EN-MINI-PRISM Analysis Tool, which is a tool for automated analysis included in the EN-MINI Tools resources [25]. We shared detailed data with representatives from the Ethiopian MoH between December 2023 and May 2024, who then discussed and validated them based on their internal datasets. Subsequently, we held a larger data validation workshop in Addis Ababa in May 2024 to discuss IMPULSE study phase 1 findings and its implications, after which priority areas for interventions and further monitoring were discussed in several follow up meetings.

The tabular format utilised to synthesise and present the PRISM indicators considered was co-created in collaboration between members of the Ethiopia and Uganda teams (FA, DF, DS), a senior author (ML), and two statisticians (LGC, IM), and was sent for revision to the whole IMPULSE study group. Two researchers (LGC, DF) checked the final structure against the structure of the PRISM Users’ Kit [25] (Table S6 in the **Online Supplementary Document**). An experienced statistician (LGC) performed the data analysis, with checks by a second statisticians (IM). All analyses were conducted in *R*, version 4.1.1 (R Foundation for Statistical Computing, Vienna, Austria).

Questions on end-users’ perspectives were analysed descriptively. Percentages were calculated separately for each tool, for site level (aggregated *via* weighted average at facility level, and at the subnational and MoH levels), and for the overall sample.

### Ethical aspects

We performed all data collection procedures in alignment with General Data Protection Regulation standards and in adherence with the principles of the Declaration of Helsinki. We did not collect any identifiable information from our participants, and we securely transmitted and stored all their data on password-protected tablets and encrypted servers, with paper documents kept in locked cabinets. We collected most data through direct observation, and conducted staff interviews only after informing them of the study’s objectives and methods, and after obtaining their written informed consent.

## RESULTS

The sample included 35 sites: 24 facilities, 10 health offices (comprising six DHOs and four regional health bureaus), and the MoH (**Table 1**). We did not include the results from central MoH (Table S7–12 in the **Online Supplementary Document**) in the synthesis figure, given that its data presents only one observation for most tools. Thus, indicators at the MoH level were expressed as the complete presence or absence of the assessed feature, with the exception of variable on tool 6, which assessed respondents’ competences in more than one person (n = 2). End users’ perspective on need for improvement from MoH were aggregated with subnational data.

**Table 1:** Sample characteristics, n (%)

Facility type (n = 35)	
Central health data office	1 (2.9)
Regional health data office	4 (11.4)
DHO	6 (17.1)
Third level of referral (national or regional) hospital	3 (8.6)
Second level of referral hospital	10 (28.6)
First level of referral health facility	11 (31.4)
**Region (n = 35)**	
Addis Ababa City Administration	5 (14.3)
Amhara and Gambela	5 (14.3)
Oromia	12 (34.3)
South Ethiopia and Sidama	13 (37.1)
**Setting – health facility only (n = 24)**	
Urban	18 (75.0)
Rural	6 (25.0)
**Managing authority– health facility only (n = 24)**	
Government/public	18 (75.0)
Private for profit	5 (20.8)
Private not-for-profit	1 (4.2)
**Job title – tool 6 respondents (n = 99)**	
Provincial HMIS focal person	6 (6.1)
District RHIS focal person	6 (6.1)
Facility in-charge (management)	8 (8.1)
Facility data management staff	27 (27.2)
Clinical ward in-charge/health worker	42 (42.4)
Other	9 (9.1)
Missing	1 (1.0)

DHO – district health office, HMIS – health management information system, RHIS – routine health information system

### Availability of ENAP-recommended indicators in the RHIS

Only 6 of the 16 WHO ENAP-recommended indicators had the same definitions in the national RHIS platform (DHIS2), while five had a different definition and five were missing (**Table 2**). We identified two nationally recommended maternal and newborn indicators not in the ENAP list at the time of the study assessment: women who developed postpartum haemorrhage and treatment outcome of neonates admitted to neonatal intensive care unit.

**Table 2:** IMPULSE study: Availability of ENAP indicators in electronic systems (DHIS2)

Sixteen WHO-recommended indicators	Type	Numerator	Denominator	Full indicator
Institutional maternal mortality ratio per 100,000 deliveries	Impact	All definitions exact	All definitions exact	All definitions exact
Stillbirth rate in a health facility	Impact	All definitions exact	All definitions exact	All definitions exact
Pre-discharge neonatal mortality rate	Impact	All definitions exact	All definitions exact	All definitions exact
Proportion of LBW among livebirths	Impact	All definitions exact	All definitions exact	No exact definitions
Preterm birth (facility-based)	Impact	Indicator unavailable	All definitions exact	Indicator unavailable
Caesarean section rate	Outcome	All definitions exact	All definitions exact	All definitions exact
Postnatal care for women (facility-based)	Outcome	All definitions exact	All definitions exact	All definitions exact
Postnatal care for newborns (facility-based)	Outcome	Indicator unavailable	All definitions exact	Indicator unavailable
Newborns breastfed within one hour of birth	Outcome	Indicator unavailable	All definitions exact	Indicator unavailable
Newborn resuscitation with bag and mask	Outcome	No exact definitions	All definitions exact	No exact definitions
Premature (LBW) babies initiating KMC	Outcome	All definitions exact	All definitions exact	No exact definitions
Newborns treated for neonatal sepsis/infection	Outcome	Indicator unavailable	All definitions exact	No exact definitions
Chlorhexidine cord cleansing	Outcome	All definitions exact	All definitions exact	No exact definitions
Antenatal corticosteroid use	Outcome	Indicator unavailable	All definitions exact	Indicator unavailable
Newborns with documented birthweight	Outcome	All definitions exact	All definitions exact	Indicator unavailable
Uterotonic for prevention of post-partum haemorrhage	Outcome	All definitions exact	All definitions exact	All definitions exact

KMC – kangaroo mother care, LBW – low birth weight, WHO – World Health Organization

### Findings by PRISM domains

#### Outputs: data quality and use

Regarding data quality, we identified major gaps in the accuracy of reported data (registers *vs*. monthly reports) on all assessed indicators, even when allowing ofr a 20-percentage-point tolerance. Availability and completeness of monthly reports were very low at DHO level, except for denominator data elements (total births and live births) which were well reported (97% and 98%, respectively). In contrast, facilities reported considerably higher rates than districts for availability and completeness of monthly reports, particularly for numerator data elements.

All indicators of data use were substandard, except for the production of narrative bulletins at DHO level (100%) and facilities sharing health indicators performance reports outside the health sector (97%). The use of RHIS data for performance monitoring (30% for DHOs, 19% for facilities), performance discussions (26% for DHOs, 12% for facilities), and information use in annual plans (67% for DHOs, 64% for facilities) was low at both district and facility levels (Tables S11 and S12 in the **Online Supplementary Document**).

#### Input factors: organisational aspects

We noted key strengths in the governance domain, with strong RHIS governance practices (80–90% at the subnational level) and use of quality improvement standards (100% at subnational level). In terms of resources, access to a working internet network was high at both subnational (100%) and facility levels (92%), as was the availability of staff to compile and analyse data (83–100%). At the MoH level, there was a positively reported availability of guidelines, standard operation procedures, and documentation that outlined RHIS mission, roles, standards, such as indicator definitions, targets, and strategic RHIS planning.

We observed heterogeneity in the subdomain of promotion of information culture, with some indicators showing good attitudes, such as: commitment/support for high quality data (81% at subnational and 80% at facility level), sharing information between levels (82% at subnational and 80% at facility level) and sense of responsibility (84% at subnational and 80% at facility level). Other subdomains required attention, such as evidence-based decision making (59% of facilities and subnational offices), promotion of problem solving (75% at subnational and 69% at facility level), and rewarding good performance (62% at subnational and 66% at facility level). Findings at the MoH level showed organisational gaps in promoting an information culture. Specifically, only 50% of respondents felt the organisation supported problem solving, while 55% believed it empowered individuals.

**Figure 3:**
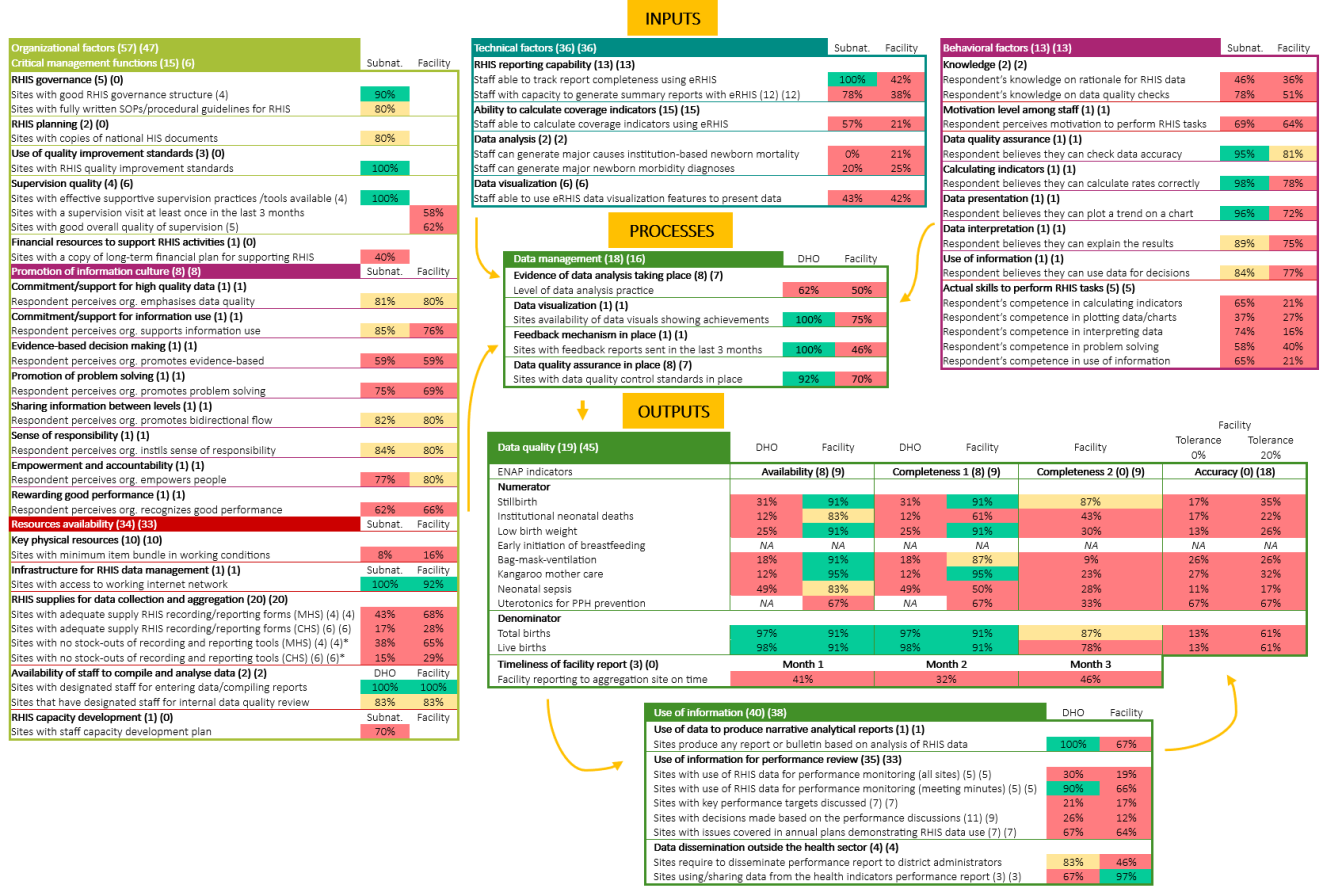
IMPULSE study synthesis figure of the EN-MINI Tools assessment based of PRISM User’s kit. Numbers in brackets next to each major and subdomain report respectively number of indicators aggregated for subnational/district data offices and facilities. Completeness 1 is specific to the monthly reports. Completeness 2 is specific to the completeness of source document. Accuracy is specific to facility level. Months 1, 2, and 3 refer to the three months of data collection. Subnational level includes regional data offices and district data offices. District level only includes district data offices. Facility level includes all three levels of healthcare facilities. Colour coding according to PRISM in green the percentages are marked between 90% and 100%, in yellow those between 80% and 89%, and in red those below 80%. Formulas are reported in Tables S7–12 in the **Online Supplementary Document**. CHS – child health services, DHO – district health office, ENAP – Every Newborn Action Plan, EN-MINI – Every Woman Every Newborn-Measurement Improvement for Newborn and Stillbirth Indicators, eRHIS – electronic routine health information system, MHS – maternal health services, NA – not applicable, org – organisation, PPH – post-partum haemorrhage, PRISM – Performance of Routine Information System Management, RHIS – routine health information system, SOPs – standard operating procedures, subnat – subnational offices. *In the last six months.

Among the recorded organisational aspects (**Figure 3**; Table S7 in the **Online Supplementary Document**), the most critical areas were the supervision quality at the facility level, both in terms of occurrence (58% of sites visited at least once in the last three months) and adherence to good practices (62% of sites reported all key elements based on a five-item criterion). We also observed significant gaps in the lack of both long-term financial plans to support RHIS (40%) and capacity development plan (70%) at subnational level.

#### Input factors: technical aspects

Tracking report completeness *via* electronic RHIS was excellent (100%) at subnational level. Staff at the MoH level demonstrated capacity to generate summary reports, calculate coverage indicators and data visualisation using eRHIS (Table S8 in the **Online Supplementary Document**). The other indicators in this domain at facility and subnational levels showed significant gaps, particularly in the ability to calculate coverage indicators, conduct data analysis, and produce data visualisations, with all showing rates <60% across all levels (**Figure 3**).

#### Input factors: behavioural aspects

Behavioural factors (**Figure 3**; Table S9 in the **Online Supplementary Document**) showed some strengths at subnational level in terms of data quality assurance (95%), calculating indicators (98%), and data presentation (96%). However, we noted a lack of respondents’ knowledge regarding data quality checks (58% at MoH level, 46% at subnational sites, 36% at facilities) and RHIS rationale (56% at MoH level, 78% at subnational sites, 51% at facilities), as well as limited motivation among staff at all levels (69% at MoH level, 69% at subnational level, 64% at facilities). Although respondents’ perceptions of their ability to perform practical tasks were high at the MoH level (88–95%) and subnational sites (84–98%), and nearly satisfactory at facilities (72–81%), a significant gap emerged when compared to their actual skills (Figure S3 in the **Online Supplementary Document**). In fact, none of the skills tested reached a satisfactory performance, although skills were better at subnational (37% to 74%) than at facility level (16% to 40%). However, at the MoH level, the gaps between skill assessed and confidence to perform RHIS activities were low in calculating indicators (12%), data presentation and interpretation (7%) and data use (5%).

#### Process: data management

Data management indicators (Table S10 in the **Online Supplementary Document**) highlighted strong data visualisation (100%), feedback mechanism (100%), and data quality assurance practices (92%) in DHOs, while the same indicators were substandard at 75%, 46%, and 70%, respectively, at the facility level. We observed a weak level of data analysis practices in both DHOs (62%) and facilities (50%).

### End users’ perspective

Answers from 252 individuals, 174 from facilities and 78 from district or higher levels captured their perspectives on the need for improvement in the existing RHIS for newborn and stillbirth care by EN-MINI domains/tools (Figure S2 and Table S5 in the **Online Supplementary Document**). Overall, an average of 57.4% of staff reported a need for any improvement across the six domains/tools, with some variations. For several domains, staff views aligned with our findings on observed indicators underlying structural weakness in the results presented so far. For example, the domains where most staff reported a need for improvement where the domains of management at subnational data offices/MoH level (72.7%) and organisational/behavioural, both at facility level (83.3%) and at subnational data offices/MoH level (80.9%). Staff responses suggested poor awareness in other domains. For example, a relatively low percentage of staff reported a need for improvement in the domain of data quality and use – 20.8% at the facility level and 16.7% at the subnational data offices and MoH.

## DISCUSSION

Here, we assessed the newborn and stillbirth data quality, use, and its determinants in Ethiopia. While there were significant strengths in governance and adherence to quality improvement standards, data quality, particularly data accuracy, was poor for all newborn indicators and limited data use for performance reviews was observed at both facility and district levels. Despite high internet access and the presence of designated staff for the RHIS, other critical weaknesses existed, including a lack of long-term financial planning at subnational sites, inadequate supplies of essential recording and reporting tools, a need for targeted training, and a need for improving supervision and staff skills at facility level in managing RHIS tasks. Other IMPULSE publications report comparison of these findings across the four IMPULSE countries [26–32]. Briefly, results from Ethiopia were showing better average performance than in other countries [27–32].

Despite these results filling a gap in existing evidence, they align with those of previous, but less comprehensive studies. Research in Ethiopia reported variability in definitions of neonatal mortality indicators within the country and emphasised the need to adopt standardised international indicator definitions to improve data comparability and usability [13]. Other investigations found that a wrong use of existing guidance on indicators definition was a technical issue in Ethiopia that adds to the data quality challenges faced nationally [13,33–35].

Regarding input and process factors, a mixed methods study across four regions in Ethiopia identified inadequate resources, infrequent supportive supervision, and gaps in staff skill and knowledge affected data quality and use in health centres and health posts [34]. A systematic review covering nine regions in Ethiopia highlighted that supportive supervision and training improved data quality, while poor registration practices, lack of commitment, and inadequate performance reviews hindered it [35]. Studies in the Amhara region and Diredawa showed that complex RHIS formats lowered data quality, while simplified ones, alongside organisational support, had the opposite effect [36,37]. In the Harari region, only 21.6% of participants had a good understanding of the rational use of RHIS data, while 77.5% received supervision, with only 14.9% receiving training [17]. Many of these factors affecting data quality and use interlink and are not easy to solve in a limited time frame [18,34–38].

As for data quality, several previous studies, although not specific to the newborn field, reported findings similar to ours [37–41]. A study in Amhara on maternal and child health indicators showed that only 74% of facilities had accurate data, while 70% of the data elements were complete [37]. In another study in Southern Ethiopia, only 44% of facilities met an acceptable accuracy level, with an overall completeness of 83.3% [39]. The results of a study in 25 health centres in Addis Ababa align with ours, with the authors reporting an overall data accuracy score of 69.6% and a completeness score of 49.5% [40]. Furthermore, a previous PRISM assessments showed that only 16% of health centres in Addis Ababa reported data within an acceptable accuracy range of 90–110%, with 42% providing timely reports [41]. Notably, according to the Ethiopia Health Information System Strategic Plan (2018–2025), the expected performance measure for quality dimensions of health data, including completeness, timeliness, and accuracy, should be greater than 90% [37]. Gaps in data accuracy may be due to multiple concomitant reasons, including the existence of multiple paper-based registers complicating aggregation, calculation and transcription issues, and DHIS2 data entry errors.

PRISM-based assessments in Ethiopia have consistently identified gaps in the use of routine health information for evidence-based decision-making despite improvements in RHIS capacity and enabling factors [27,41]. Other research tested interventions to increase the frequency of performance monitoring team meetings already established in 24 facilities, but only two-thirds conducted the meetings regularly [27,41]. A cross-sectional study in Diredawa found that only 57.7% of healthcare professionals effectively utilised data [36], while a study in the Oromia region reported that 71.6% of routine health data was used for decision making [38]. Additionally, a qualitative study highlighted that both data use for programming and the overall culture of data use were not fully developed [34].

Ethiopia has already introduced strategic interventions to improve data quality and use in RHIS [27,32,41], with the most prominent being the Information Revolution Strategic Plan [37], which included the cultural transformation of the information system to bring fundamental change to the Ethiopian health information system. Furthermore, the Ministry of Health, regional health bureaus, and six local universities created a capacity-building programme which offered technical support to health information systems of governing bodies [32,41,42]. Several studies indicated that these initiatives enhanced the Ethiopian RHIS [32,40,41].

### Strengths and limitations

Ours is one of the few studies reporting a comprehensive assessment of 378 indicators of newborn and stillbirth data quality and use, and the underlying determinants, according to a predefined PRISM framework. We collected most of our data through direct observation, with several quality assurance procedures in place. We also used a standardised methodology for data collection that can be easily replicated in other settings and allows for further monitoring in the context of our sample. Our findings can drive quality improvement initiatives and provide data that gives attention to the newborn and stillbirth data quality and use gaps and the complex factors that result in poor data quality and utilisation. Lastly, the end user’s perspective collected in our analysis further contribute to the identification of critical areas for intervention with practical and actionable targets.

We acknowledge that our findings are not directly generalisable to the entire country. The selection of comprehensive emergency obstetric emergency and newborn care facilities and the inclusion of conflict regions, as well as data collection period, might have led to biases in our estimates. We did not collect sociodemographic information from end-users; however, we followed the EN-MINI manual in selecting them and key staff from service delivery sites and offices. In relation to external limitations, some gaps in the availability and completeness of numerator indicators may stem from the Ethiopian DHIS-2 system’s inability to differentiate between unreported data and zero-reported cases at district level, which is a limitation beyond the control of our study. Lastly, the PRISM methodology [25], although having the merit of exploring many indicators, lacks composite predefined summary indicators enabling assessing performance for each major domain, thus hampering multivariate analyses and further inference into potential casual factors. While the PRISM methodology, which aggregates groups of indicators into average percentages, is widely used, it has important limitations, particularly the potential masking of variation between individual indicators. Nevertheless, it remains consistent with standard practice in comparable assessments, largely due to the lack of empirical justification for assigning differential weights to indicators.

## CONCLUSIONS

We found significant strengths and gaps in newborn and stillbirth data quality and use in RHIS in Ethiopia, as assessed through direct observation with the PRISM methodology. Overall, we suggest several key areas for intervention to enhance the quality and use of newborn and stillbirth data our study sites: data accuracy, development of staff capacities, and targeted and tailored supporting supervision. Some of these challenges will be addressed by IMPULSE phase II after discussion with local stakeholders; other, more structural ones should be addressed by wider health system strengthening interventions that require, major effort, including routine monitoring and long-term evaluation.

## Additional material


Online Supplementary Document
