# Supplementary material for: Stillbirth and newborn data quality and use and related input and process factors: findings of the IMPULSE study in Ethiopia
Source: J Glob Health. 2026 Jul 17;16:04231. doi: 10.7189/jogh.16.04231 (PMC13377767; doi:10.7189/jogh.16.04231)
Supplement: Online Supplementary Document [file jogh-16-04231-s001.pdf]

Supplement to: Fisseha D, Abathun F, Cora LG, Worku B, Belgu B, Girmay AM, Chimuna T, Solomon M, Abebe H, Mariani I, Ayele M, Rornald MK, Minja J, Mouhamadou O, Tognon F, Lawn JE, Putoto G, Shamba D, Waiswa P, Awell T, Lazzerini M. Stillbirth and newborn data quality and use and related input and process factors: findings of the IMPULSE study in Ethiopia. J Glob Health. 2026;16:04231.

TABLES

Table S1. The Strengthening the Reporting of Observational Studies (STROBE) Checklist. 1

Table S2. Sampling criteria. 2

Table S3. Characteristics of the regions. 3

Table S4. Sample size by tool and facility type. 4

Table S5. End users' perspectives (N = 252). 5

Table S6. Cross checks with PRISM User's kit. 6

Table S7. Organizational factors. 7

Table S8. Technical factors. 9

Table S9. Behavioral factors. 10

Table S10. Data management. 11

Table S11. Data quality. 12

Table S12. Data use. 17

Table S13. Number of indicators in each major category by facility type. 18

FIGURES

Figure S1. PRISM Framework and conceptual model. 19

Figure S2. IMPULSE Study End users’ perspectives on the need for improvement in RHIS (252 respondents). 20

Figure S3. Gap between respondents' competence and confidence in carrying out RHIS tasks. 21

TABLES

Table S1. The Strengthening the Reporting of Observational Studies (STROBE) Checklist.

	Item No	Recommendation	Pages
**Title and abstract**	1	(*a*) Indicate the study's design with a commonly used term in the title or the abstract	1
		(*b*) Provide in the abstract an informative and balanced summary of what was done and what was found	1
**Introduction**			**3**
Background/rationale	2	Explain the scientific background and rationale for the investigation being reported	3
Objectives	3	State specific objectives, including any prespecified hypotheses	3
**Methods**			**4**
Study design	4	Present key elements of study design early in the paper	4
Setting	5	Describe the setting, locations, and relevant dates, including periods of recruitment, exposure, follow-up, and data collection	4
Participants	6	(*a*) Give the eligibility criteria, and the sources and methods of selection of participants	4
Variables	7	Clearly define all outcomes, exposures, predictors, potential confounders, and effect modifiers. Give diagnostic criteria, if applicable	4-6
Data sources/ measurement	8*	For each variable of interest, give sources of data and details of methods of assessment (measurement). Describe comparability of assessment methods if there is more than one group	5-6
Bias	9	Describe any efforts to address potential sources of bias	6
Study size	10	Explain how the study size was arrived at	6
Quantitative variables	11	Explain how quantitative variables were handled in the analyses. If applicable, describe which groupings were chosen and why	6-8
Statistical methods	12	(*a*) Describe all statistical methods, including those used to control for confounding	6-8
		(*b*) Describe any methods used to examine subgroups and interactions	6-8
		(*c*) Explain how missing data were addressed	6-8
		(*d*) If applicable, describe analytical methods taking account of sampling strategy	6-8
		(*e*) Describe any sensitivity analyses	6-8
**Results**			**9**
Participants	13*	(a) Report numbers of individuals at each stage of study—e.g. numbers potentially eligible, examined for eligibility, confirmed eligible, included in the study, completing follow-up, and analysed	9
		(b) Give reasons for non-participation at each stage	
		(c) Consider use of a flow diagram	
Descriptive data	14*	(a) Give characteristics of study participants (e.g. demographic, clinical, social) and information on exposures and potential confounders	9
		(b) Indicate number of participants with missing data for each variable of interest	8-9
Outcome data	15*	Report numbers of outcome events or summary measures	8-9
Main results	16	(*a*) Give unadjusted estimates and, if applicable, confounder-adjusted estimates and their precision (e.g., 95% confidence interval). Make clear which confounders were adjusted for and why they were included	10-14
		(*b*) Report category boundaries when continuous variables were categorized	
		(*c*) If relevant, consider translating estimates of relative risk into absolute risk for a meaningful time period	
Other analyses	17	Report other analyses done—e.g. analyses of subgroups and interactions, and sensitivity analyses	
**Discussion**			**14**
Key results	18	Summarise key results with reference to study objectives	14-16
Limitations	19	Discuss limitations of the study, taking into account sources of potential bias or imprecision. Discuss both direction and magnitude of any potential bias	14-16
Interpretation	20	Give a cautious overall interpretation of results considering objectives, limitations, multiplicity of analyses, results from similar studies, and other relevant evidence	14-16
Generalisability	21	Discuss the generalisability (external validity) of the study results	14-16
**Other information**			**17**
Funding	22	Give the source of funding and the role of the funders for the present study and, if applicable, for the original study on which the present article is based	17

*give information separately for exposed and unexposed groups.

Table S2. Sampling criteria.

**Facility type**	**Criteria**
Health facilities	
In each country	
3rd level of referral (National)	1
In each Region	
3rd level of referral (Regional)	1
2nd level of referral (Subnational / District) Public	2
2nd level of referral (Subnational / District) Not for Profit	1 (if existing, and allowing)
2nd level of referral (Subnational / District) Private	1 (if existing, and allowing)
1st level of referral (Primary Hospital / Health Center with CEmONC) Public	3
1st level of referral (Primary Hospital / Health Center with CEmONC) Private	1-2 (if existing, and allowing)
Data offices	
District /Subnational health office	all data offices related to selected facilities
Regional health office	yes
National data office	yes

BEmONC = basic emergency obstetric and neonatal care, CEmONC = comprehensive emergency obstetric and newborn care.
Further details are presented in **Figure 2**.

Table S3. Characteristics of the regions.

**Major characteristic**	Addis Ababa City Administration		Amhara		Gambela		Oromia		South Ethiopia		Sidama	
	N	%	N	%	N	%	N	%	N	%	N	%
Facility type	N = 5		N = 2		N = 3		N = 12		N = 9		N = 4	
Central health data office	1	20	NA	NA	NA	NA	NA	NA	NA	NA	NA	NA
Regional health data office	1	20	0	0	1	33.3	1	8.3	0	0	1	25
District health data office	0	0	1	50	0	0	2	16.7	3	33.3	0	0
3rd level referral (national or regional)	1	20	0	0	0	0	1	8.3	1	11.1	0	0
2nd level of referral	2	40	1	50	1	33.3	3	25	2	22.2	1	25
1sr level of referral	0	0	0	0	1	33.3	5	41.7	3	33.3	2	50
Setting	N = 3		N = 1		N = 2		N = 9		N = 6		N = 3	
Rural	0	0	0	0	0	0	3	33.3	3	50	0	0
Urban	3	100	1	100	2	100	6	66.7	3	50	3	100
Managing authority	N = 5		N = 2		N = 3		N = 12		N = 9		N = 4	
Government/public	5	100	2	100	3	100	9	75	9	100	1	25
Private for profit	0	0	0	0	0	0	2	16.7	0	0	3	75
Private not-for-profit	0	0	0	0	0	0	1	8.3	0	0	0	0
Hard to reach/disadvantaged area	No		No		No		No		Yes (semi nomadic population, flood risk)		Yes (semi nomadic population, flood risk)	
Humanitarian setting	No		No		Yes (refugees)		No		No		No	

NA = not applicable.

Table S4. Sample size by tool and facility type.

**Sample size by tool**	N	%
Tool 1 - RHIS Overview (RHIS)	N = 35	
Central health data office	1	2.9
Regional health data office	4	11.4
District health data office	6	17.1
Third level of referral (national or regional) hospital	3	8.6
Second level of referral hospital	10	28.6
First level of referral health facility	11	31.4
Tool 2A - RHIS Performance diagnosis district level	N = 6	
Central health data office	NA	NA
Regional health data office	6	100
District health data office	NA	NA
Third level of referral (national or regional) hospital	NA	NA
Second level of referral hospital	NA	NA
First level of referral health facility	NA	NA
Tool 2B - RHIS Performance diagnosis health facility level (DQ-DU-FQ-FU)	N = 24	
Central health data office	NA	NA
Regional health data office	NA	NA
District health data office	NA	NA
Third level of referral (national or regional) hospital	3	12.5
Second level of referral hospital	10	41.7
First level of referral health facility	11	45.8
Tool 3.1 - eRHIS functionality assessment (ESF)	N = 8	
Central health data office	1	12.5
Regional health data office	4	50
District health data office	3	37.5
Third level of referral (national or regional) hospital	NA	NA
Second level of referral hospital	NA	NA
First level of referral health facility	NA	NA
Tool 3.2 - eRHIS usability assessment (ESU)	N = 35	
Central health data office	1	2.9
Regional health data office	4	11.4
District health data office	6	17.1
Third level of referral (national or regional) hospital	3	8.6
Second level of referral hospital	10	28.6
First level of referral health facility	11	31.4
Tool 4 - Management assessment (MAT)	N = 11	
Central health data office	1	9.1
Regional health data office	4	36.4
District health data office	6	54.5
Third level of referral (national or regional) hospital	NA	NA
Second level of referral hospital	NA	NA
First level of referral health facility	NA	NA
Tool 5 - Facility/office assessment (FOC)	N = 34	
Central health data office	NA	NA
Regional health data office	4	11.8
District health data office	6	17.6
Third level of referral (national or regional) hospital	3	8.8
Second level of referral hospital	10	29.4
First level of referral health facility	11	32.4
Tool 6 - Organizational/behavioral assessment *(OBAT)	N = 99	
Central health data office	2	2
Regional health data office	8	8.1
District health data office	11	11.1
Third level of referral (national or regional) hospital	9	9.1
Second level of referral hospital	32	32.3
First level of referral health facility	37	37.4

*data from Tool 6 are expressed in terms of respondents rather than number of sites.
In brackets are reported the codification for the PRISM User's Kit [25].
NA = not applicable.

Table S5. End users' perspectives (N = 252).

**EN MINI Tool**	**Facility**								**Subnational + MoH**								**Overall**							
	Major		Some		No		Any improvement		Major		Some		No		Any improvement		Major		Some		No		Any improvement	
	N	%	N	%	N	%	N	%	N	%	N	%	N	%	N	%	N	%	N	%	N	%	N	%
Tool 1 - RHIS Overview	N = 24								N = 11								N = 35							
(data collection, information systems mapping and flow)	2	8.3	5	20.8	17	70.8	7	29.2	2	18.2	2	18.2	7	63.6	4	36.4	4	11.4	7	20	24	68.6	11	31.4
Tool 2 - RHIS Performance Diagnostic	N = 24								N = 6								N = 30							
(data quality/use)	1	4.2	4	16.7	19	79.2	5	20.8	0	0	1	16.7	5	83.3	1	16.7	1	3.3	5	16.7	24	80	6	20
Tool 3.1 - eRHIS Functionality	NA								N = 8								N = 8							
(data integration, disaggregation, analysis, visualization; report generation)	NA	NA	NA	NA	NA	NA	NA	NA	2	25	1	12.5	5	62.5	3	37.5	2	25	1	12.5	5	62.5	3	37.5
Tool 3.2 - eRHIS Usability	N = 24								N = 11								N = 35							
	2	8.3	5	20.8	17	70.8	7	29.2	3	27.3	3	27.3	5	45.5	6	54.5	5	14.3	8	22.9	22	62.8	13	37.1
Tool 4 - Management	NA								N = 11								N = 11							
	NA	NA	NA	NA	NA	NA	NA	NA	4	36.4	4	36.4	3	27.3	8	72.7	4	36.4	4	36.4	3	27.3	8	72.7
Tool 5 - Facility/Office Assessment	N = 24								N = 10								N = 34							
(resources)	4	16.7	13	54.2	7	29.2	17	70.8	3	30	4	40	3	30	7	70	7	20.6	17	50	10	29.4	24	70.6
Tool 6 - Organizational/Behavioral	N = 78								N = 21								N = 99							
	15	19.2	50	64.1	13	16.7	65	83.3	4	19	13	61.9	4	19	17	80.9	19	19.2	63	63.6	17	17.1	82	82.8
Weighted average	24	13.8	77	44.3	73	41.9	101	58.0	14	20.6	24	35.3	29	42.6	38	55.9	38	15.7	101	41.7	102	42.1	139	57.4

eRHIS = electronic routine health information system, MoH = Ministry of Health, NA = not applicable, RHIS = routine health information system.

Table S6. Cross checks with PRISM User's kit [25].

PRISM Users' kit contents	Synthesis
Use of information	
II. RHIS Performance: Use of Information Indicators .... 32<br>A. Use of Data to Produce Narrative Analytical Reports .... 32<br>B. Use of Information for Performance Review .... 33<br>C. Data Dissemination outside the Health Sector 40	**Use of information (40) (38)**<br>**Use of data to produce narrative analytical reports (1) (1)**<br>Sites produce any report or bulletin based on analysis of RHIS data<br>**Use of information for performance review (35) (33)**<br>Sites with use of RHIS data for performance monitoring (all sites) (5) (5)<br>Sites with use of RHIS data for performance monitoring (meeting minutes) (5) (5)<br>Sites with key performance targets discussed (7) (7)<br>Sites with decisions made based on the performance discussions (11) (9)<br>Sites with issues covered in annual plans demonstrating RHIS data use (7) (7)<br>**Data dissemination outside the health sector (4) (4)**<br>Sites require to disseminate performance report to district administrators<br>Sites using/sharing data from the health indicators performance report (3) (3)
Data management	
III. RHIS Performance: Data Management Indicators .... 42<br>A. Data Quality Assurance System in Place .... 42<br>B. Evidence of Data Analysis Taking Place .... 45<br>C. Data Visualization .... 47<br>D. Feedback Mechanism in Place 48	**Data management (18) (16)**<br>**Evidence of data analysis taking place (8) (7)**<br>Level of data analysis practice<br>**Data visualization (1) (1)**<br>Sites availability of data visuals showing achievements<br>**Feedback mechanism in place (1) (1)**<br>Sites with feedback reports sent in the last 3 months<br>**Data quality assurance in place (8) (7)**<br>Sites with data quality control standards in place
Technical factors	
IV. RHIS Performance Determinants: Technical Factors .... 49<br>A. Existing Information System Overlaps and Distinctions .... 49<br>B. Standardization of RHIS Tools .... 50<br>C. eRHIS Reporting Capability .... 52<br>D. Population Estimates and Coverage .... 53<br>E. System Captures Age and Sex-Disaggregated Data .... 53<br>F. Data Integration and Interoperability .... 54<br>G. Unique Identifiers and Master Facility List .... 55<br>H. Data Analysis .... 55<br>I. Data Visualization .... 56<br>J. RHIS Reporting Capability .... 57<br>K. Ability to Calculate Coverage Indicators .... 58<br>L. Data Analysis .... 59<br>M. Data Visualization 59	**Technical factors (36) (36)**<br>**RHIS reporting capability (13) (13)**<br>Staff able to track report completeness using eRHIS<br>Staff with capacity to generate summary reports with eRHIS (12) (12)<br>**Ability to calculate coverage indicators (15) (15)**<br>Staff able to calculate coverage indicators using eRHIS<br>**Data analysis (2) (2)**<br>Staff can generate major causes institution-based newborn mortality<br>Staff can generate major newborn morbidity diagnoses<br>**Data visualization (6) (6)**<br>Staff able to use eRHIS data visualization features to present data
Organizational and behavioral factors	
V. RHIS Performance Determinants: Organizational Factors .... 60<br>A. RHIS Governance .... 60<br>B. RHIS Planning .... 62<br>C. Use of Quality Improvement Standards .... 62<br>D. Supervision Quality .... 63<br>E. Financial Resources to Support RHIS Activities .... 65<br>F. Infrastructure for RHIS Data Management .... 65<br>G. RHIS Supplies for Data Collection and Aggregation .... 66<br>H. Availability of Staff to Compile and Analyze Data .... 68<br>I. RHIS Capacity Development .... 73<br>J. Commitment to and Support for High-Quality Data .... 76<br>K. Commitment to and Support for Information Use .... 77<br>L. Evidence-Based Decision Making .... 78<br>M. Promotion of Problem Solving .... 80<br>N. Sharing Information between Levels .... 80<br>O. Sense of Responsibility .... 81<br>P. Empowerment and Accountability .... 82<br>Q. Rewarding Good Performance .... 83<br>R. Data Quality Assurance .... 83<br>S. Calculating Indicators .... 84<br>T. Data Presentation .... 84<br>U. Data Interpretation .... 85<br>V. Use of Information .... 85<br>W. Motivation Level among Staff .... 86<br>X. Knowledge .... 87<br>Y. Actual Skills to Perform RHIS Tasks 90	**Organizational factors (57) (47)**<br>**Critical management functions (15) (6)**<br>**RHIS governance (5) (0)**<br>Sites with good RHIS governance structure (4)<br>Sites with fully written SOPs/procedural guidelines for RHIS<br>**RHIS planning (2) (0)**<br>Sites with copies of national HIS documents<br>**Use of quality improvement standards (3) (0)**<br>Sites with RHIS quality improvement standards<br>**Supervision quality (4) (6)**<br>Sites with effective supportive supervision practices /tools available (4)<br>Sites with a supervision visit at least once in the last 3 months<br>Overall quality of supervision (5)<br>**Financial resources to support RHIS activities (1) (0)**<br>Sites with allocated financial resources for RHIS activities<br>**Promotion of information culture (8) (8)**<br>**Commitment/support for high quality data (1) (1)**<br>Respondent perceives org. emphasises data quality<br>**Commitment/support for information use (1) (1)**<br>Respondent perceives org. supports information use<br>**Evidence-based decision making (1) (1)**<br>Respondent perceives org. promotes evidence-based<br>**Promotion of problem solving (1) (1)**<br>Respondent perceives org. promotes problem solving<br>**Sharing information between levels (1) (1)**<br>Respondent perceives org. promotes bidirectional flow<br>**Sense of responsibility (1) (1)**<br>Respondent perceives org. instils sense of responsibility<br>**Empowerment and accountability (1) (1)**<br>Respondent perceives org. empowers people<br>**Rewarding good performance (1) (1)**<br>Respondent perceives org. recognizes good performance<br>**Resources availability (34) (33)**<br>**Key physical resources (10) (10)**<br>Sites with minimum item bundle in working conditions<br>**Infrastructure for RHIS data management (1) (1)**<br>Sites with access to working internet network<br>**RHIS supplies for data collection and aggregation (20) (20)**<br>Sites with adequate supply RHIS recording/reporting forms (MHS) (4) (4<br>Sites with adequate supply RHIS recording/reporting forms (CHS) (6) (6)<br>Sites with no stock-outs of recording and reporting tools (MHS) (4) (4)*<br>Sites with no stock-outs of recording and reporting tools (CHS) (6) (6)*<br>**Availability of staff to compile and analyse data (2) (2)**<br>Sites with designated staff for entering data/compiling reports<br>Sites that have designated staff for internal data quality review<br>**RHIS capacity development (1) (0)**<br>Sites with staff capacity development plan<br><br>**Behavioral factors (13) (13)**<br>**Knowledge (2) (2)**<br>Respondent's knowledge on rationale for RHIS data<br>Respondent's knowledge on data quality checks<br>**Motivation level among staff (1) (1)**<br>Respondent perceives motivation to perform RHIS tasks<br>**Data quality assurance (1) (1)**<br>Respondent believes they can check data accuracy<br>**Calculating indicators (1) (1)**<br>Respondent believes they can calculate rates correctly<br>**Data presentation (1) (1)**<br>Respondent believes they can plot a trend on a chart<br>**Data interpretation (1) (1)**<br>Respondent believes they can explain the results<br>**Use of information (1) (1)**<br>Respondent believes they can use data for decisions<br>**Actual skills to perform RHIS tasks (5) (5)**<br>Respondent's competence in calculating indicators<br>Respondent's competence in plotting data/charts<br>Respondent's competence in interpreting data<br>Respondent's competence in problem solving<br>Respondent's competence in use of information

CHS = child health services, eRHIS = electronic health information system, MHS = mother health services, PRISM = performance of routine information system management, RHIS = routine health information system.

Table S7. Organizational factors.

Organizational factors		PRISM formula		Regional data offices			District data offices			Facilities			Subnational*			MoH		
Critical managment functions	Description indicator	Numerator	Denominator	Numerator	Denominator	Percentage	Numerator	Denominator	Percentage	Numerator	Denominator	Percentage	Numerator	Denominator	Percentage	Numerator	Denominator	Presence/Absence
**RHIS governance**																		
Sites with good RHIS governance structure	Has written document describing the RHIS mission, roles, and responsibilities that are related to strategic and policy decisions at the district and higher levels	MAT005	Number of districts assesed	3	4	75%	5	6	83%				8	10	80%	1	1	Presence
	Has current health service organizational and staff chart showing positions related to health information	MAT006	Number of districts assesed	3	4	75%	6	6	100%				9	10	90%	1	1	Presence
	Office has an overall framework and plan for information and communication technology (ICT), for example, describing the required equipment and plans for training in the use of ICT for RHIS	MAT008	Number of districts assesed	3	4	75%	6	6	100%				9	10	90%	1	1	Presence
	Office maintains a list/documentation of the dissemination of the RHIS monthly/quarterly reports to the various health program staff in the district, the community, local administration, nongovernmental organizations (NGOs), etc.	MAT009	Number of districts assesed	4	4	100%	6	6	100%				10	10	100%	0	1	Absence
	Aggregated indicator	Sum of numerators	4*Number of districts assessed	13	16	81%	23	24	96%				36	40	90%			
Sites with fully written SOPs/procedural guidelines for RHIS	Office has written standard operating procedures (SOPs) and procedural guidelines for the RHIS that include data definitions; data collection and reporting; data aggregation, processing, and transmission; data analysis, dissemination, and use; data quality assurance; MFL; International Classification of Disease (ICD) codes; data security; data storage; and performance improvement processes	MAT007a=1	Number of districts assesed	3	4	75%	5	6	83%				8	10	80%	1	1	Presence
**RHIS planning**																		
Sites with copies of national HIS documents	Office has a copy of the national HIS situation analysis/assessment report that is less than three years old	MAT010	Number of districts assesed	2	4	50%	4	6	67%				6	10	60%	1	1	Presence
	Office has a copy of the national three- or five-year HIS strategic plan	MAT011	Number of districts assesed	4	4	100%	6	6	100%				10	10	100%	1	1	Presence
	Aggregated indicator	Sum of numerators	2*Number of districts assessed	6	8	75%	10	12	83%				16	20	80%			
**Use of quality improvements standards**																		
Sites with RHIS quality improvements standards	Has set RHIS performance targets RHIS performance targets for data accuracy for their respective administrative areas	MAT012.1	Number of districts assesed	4	4	100%	6	6	100%				10	10	100%	0	1	Absence
	Has set RHIS performance targets RHIS performance targets for data completeness for their respective administrative areas	MAT012.2	Number of districts assesed	4	4	100%	6	6	100%				10	10	100%	1	1	Presence
	Has set RHIS performance targets RHIS performance targets for data timeliness for their respective administrative areas	MAT012.3	Number of districts assesed	4	4	100%	6	6	100%				10	10	100%	1	1	Presence
	Aggregated indicator			12	12	100%	18	18	100%				10	30	100%			
**Supervision quality**																		
Sites with effective supportive supervision practices /tools available	Office has copies of RHIS supervisory guidelines and checklists	MAT018	Number of districts assesed	4	4	100%	6	6	100%				10	10	100%	0	1	Absence
	Office maintains a schedule for RHIS supervisory visits	MAT019	Number of districts assesed	4	4	100%	6	6	100%				10	10	100%	1	1	Presence
	Office has copies of the reports from RHIS supervisory visits conducted during the current fiscal year	MAT020	Number of districts assesed	4	4	100%	6	6	100%				10	10	100%	0	1	Absence
	HFs that received a supervisory visit have copies of the report from latest supervisory visit and commonly agreed action points are listed	MAT021	Number of districts assesed	4	4	100%	6	6	100%				10	10	100%	1	1	Presence
	Aggregated indicator	Sum of numerators	4*Number of districts assessed	16	16	100%	24	24	100%				40	10	100%			
Sites with a supervision visit at least oncce in the last 3 months	Frequency of facilities that received at least one district supervisor's visit(s)over the past three months	FU022	Number of facilities assesed							10	24	58%						
Overall quality of supervision	Supervisor checked the data quality	FU023	Number of facilities with at least 1 visit (FU022=1 to 5)							7	10	70%						
	Supervisor used a checklist to assess the data quality	FU024	Number of facilities with at least 1 visit (FU022=1 to 5)							7	10	70%						
	During the visit, the district supervisor discussed the health facility's performance based on the RHIS information	FU025	Number of facilities with at least 1 visit (FU022=1 to 5)							7	10	70%						
	Supervisor helped the respondent to make a decision or to take corrective action based on the discussion	FU026	Number of facilities with at least 1 visit (FU022=1 to 5)							6	10	60%						
	Supervisor sent a report/written feedback on the last supervisory visit(s)	FU027	Number of facilities with at least 1 visit (FU022=1 to 5)							4	10	40%						
	Aggregated indicator	Sum of numerators	5*Number of facilities with at least 1 visit (FU022=1 to 5)							31	50	62%						
Sites with allocated financial resources for RHIS activities	Office has a copy of the long-term financial plan for supporting RHIS activities	MAT024	Number of districts assesed	2	4	50%	2	6	33%				4	10	40%	1	1	Presence
**Promotion of information culture**																		
**Commitment/support for high quality data**																		
Respondent perceives organization emphasise data quality	Respondent perceives that the organization gives due emphasis to data quality	S2<br>S6<br>S8	15*number of respondents	96	120	80%	134	165	81%	925	1155	80%	230	285	81%	24	30	80%
**Commitment/support for information use**																		
Respondents perceive org. supports information use	Respondent perceives that the organization supports information use	S4<br>S7<br>P5<br>P8	20*number of respondents	135	160	84%	189	220	86%	1166	1540	76%	324	380	85%	27	40	68%
**Evidence-based decision making**																		
Respondents perceive org. promotes evidence-based	Respondent perceives that the organization promotes a culture of evidence-based decision making	Inverse D1<br>Inverse D2<br>D3<br>Inverse D4<br>Inverse D5<br>Inverse D6<br>D7<br>D8<br>D9<br>D10	50*number of respondents	236	400	59%	326	550	59%	2257	3850	59%	562	950	59%	56	100	56%
**Promotion of problem solving**																		
Respondents perceive org. promotes problem solving	Respondent perceives that the organization promotes a culture of problem solving	S5<br>P6<br>P7<br>P9	20*number of respondents	120	160	75%	165	220	75%	1059	1540	69%	285	380	75%	20	40	50%
**Sharing information between levels**																		
Respondents perceive org. promotes bidirectional flow	Respondent perceives that the organization promotes a bidirectional flow of feedback	S1<br>S3	20*number of respondents	64	80	80%	92	110	84%	616	770	80%	156	190	82%	18	20	90%
**Sense of responsability**																		
Respondents perceive org. instils sense of responsibility	Respondent perceives that the organization has a culture that instills a sense of responsibility	P1<br>P2<br>P3<br>P4<br>P12	25*number of respondents	162	200	81%	235	275	85%	1543	1925	80%	397	475	84%	35	50	70%
**Empowerment and Accountability**																		
Respondents perceive org. empowers people	Respondent perceives that the organization empowers people to ask questions, seek improvement, learn, and improve quality through useful information	P10<br>P11	10*number of respondents	55	80	69%	92	110	84%	616	770	80%	147	190	77%	11	20	55%
**Rewarding Good Performance**																		
Respondents perceive org. recognizes good performance	Respondent perceives that the organization recognizes and rewards good performance	S9	5 * number of respondents	25	40	63%	34	55	62%	253	385	66%	59	95	62%	6	10	60%

Resources availability																		
Infrastructure for RHIS data management																		
Sites with access to working internet network	Access to an Internet network	FOC025	Number of sites assessed	4	4	100%	6	6	100%	22	24	92%	10	10	100%			
RHIS supplies for data collection and aggregation																		
Sites with adequate supply RHIS recording/reporting forms (MHS)	Maternal and newborn health services – Labor and delivery - printed register	FOC__032_5.1	Number of sites assessed	2	4	50%	3	6	50%	24	24	100%	5	10	50%			
	Maternal and newborn health services – Operation theater - printed register	FOC__032_5.2	Number of sites assessed	2	4	50%	3	6	50%	16	24	67%	5	10	50%			
	Maternal health services – Postnatal ward printed register	FOC__032_5.3	Number of sites assessed	2	4	50%	4	6	67%	22	24	92%	6	10	60%			
	Maternal health services – Death printed register	FOC__032_5.4	Number of sites assessed	1	4	25%	0	6	0%	3	24	13%	1	10	10%			
	Aggregated indicator	Sum of numerators	4*Number of sites assessed	7	16	44%	10	24	42%	65	96	68%	17	40	43%			
Sites with adequate supply RHIS recording/reporting forms (CHS)	Child/Newborn health services – Postnatal ward printed register	FOC_032_6.1	Number of sites assessed	1	4	25%	3	6	50%	13	24	54%	4	10	40%			
	Child/Newborn health services – Kangaroo mother care (KMC) printed register	FOC_032_6.2	Number of sites assessed	0	4	0%	0	6	0%	2	24	8%	0	10	0%			
	Child/Newborn health services – Neonatal inpatient care ward printed register	FOC_032_6.3	Number of sites assessed	0	4	0%	0	6	0%	1	24	4%	0	10	0%			
	Child/Newborn health services – Special care newborn ward printed register	FOC_032_6.4	Number of sites assessed	0	4	0%	0	6	0%	1	24	4%	0	10	0%			
	Child/Newborn health services – Neonatal Intensive Care unit (NICU) printed register	FOC_032_6.5	Number of sites assessed	2	4	50%	4	6	67%	22	24	92%	6	10	60%			
	Child/Newborn health services – Death printed register	FOC_032_6.6	Number of sites assessed	0	4	0%	0	6	0%	2	24	8%	0	10	0%			
	Aggregated indicator	Sum of numerators	6*Number of sites assessed	3	24	12%	7	36	19%	41	144	28%	10	60	17%			
Sites with no stock-outs of recording and reporting tools (MHS)	Maternal and newborn health services – Labor and delivery - printed register	FOC_034_5.1	Number of sites assessed	2	4	50%	2	6	30%	23	24	96%	4	10	40%			
	Maternal and newborn health services – Operation theater - printed register	FOC_034_5.2	Number of sites assessed	2	4	50%	3	6	50%	16	24	67%	5	10	50%			
	Maternal health services – Postnatal ward printed register	FOC_034_5.3	Number of sites assessed	2	4	50%	3	6	50%	20	24	83%	5	10	50%			
	Maternal health services – Death printed register	FOC_034_5.4	Number of sites assessed	1	4	25%	0	6	0%	3	24	13%	1	10	10%			
	Aggregated indicator	Sum of numerators	4*Number of sites assessed	7	16	44%	8	24	33%	62	96	65%	15	40	38%			
Sites with no stock-outs of recording and reporting tools (CHS)	Child/Newborn health services – Postnatal ward printed register	FOC_034_6.1	Number of sites assessed	1	4	25%	3	6	50%	14	24	58%	4	10	40%			
	Child/Newborn health services – Kangaroo mother care (KMC) printed register	FOC_034_6.2	Number of sites assessed	0	4	0%	0	6	0%	2	24	8%	0	10	0%			
	Child/Newborn health services – Neonatal inpatient care ward printed register	FOC_034_6.3	Number of sites assessed	0	4	0%	0	6	0%	1	24	4%	0	10	0%			
	Child/Newborn health services – Special care newborn ward printed register	FOC_034_6.4	Number of sites assessed	0	4	0%	0	6	0%	1	24	4%	0	10	0%			
	Child/Newborn health services – Neonatal Intensive Care unit (NICU) printed register	FOC_034_6.5	Number of sites assessed	1	4	25%	4	6	67%	22	24	92%	5	10	50%			
	Child/Newborn health services – Death printed register	FOC_034_6.6	Number of sites assessed	0	4	0%	0	6	0%	2	24	8%	0	10	0%			
	Aggregated indicator	Sum of numerators	6*Number of sites assessed	2	24	8%	7	36	19%	42	144	29%	9	60	15%			
Availability of staff to compile and analyse data																		
Sites with designated staff for entering data/compiling reports	District has a designated person responsible for entering data/compiling reports from health facilities	DQ010	Number of districts assesed				6	6	100%									
	A designated person enters data/compiles reports from the different units in the health facility	FQ0111	Number of facilities assesed							24	24	100%						
Sites that have designated staff for internal data quality review	District has a designated person to review the quality of compiled data prior to submission to the next level	DQ011	Number of districts assesed				5	6	83%									
	A designated person reviews the quality of compiled data prior to submission to the next level	FQ012	Number of facilities assesed							20	24	83%						
RHIS capacity development																		
Sites with staff capacity development plan	Office has a costed training and capacity development plan that has benchmarks, timelines, and mechanisms for on-the-job RHIS training, RHIS workshops, and orientation for new staff	MAT016	Number of districts assesed	3	4	75%	4	6	67%				7	10	70%	1	1	Presence

RHIS = routine health information system, MHS = mother health services, CHS = child health services.

*definition of the IMPULSE study; it aggregates regional data offices and district data offices.

Grey coloured portions of the table indicate that the indicator is not recorded either at district or facility level; in green are marked the indicators with a reported percentage between 90% and 100%, in yellow those between 80% and 89%, and in red those below 80%.

Table S8. Technical factors.

Technical factors		PRISM formula		Regional data offie			District data offices			Facilities			Subnational*			MoH*		
RHIS reporting capability	Description indicator	Numerator	Denominator	Numerator	Denominator	Percentage	Numerator	Denominator	Percentage	Numerator	Denominator	Percentage	Numerator	Denominator	Percentage	Numerator	Denominator	Presence/absence
Staff able to track report completeness using eRHIS	User can carry out the following function: RHIS software produces a report on the number and percentage of reports received out of the total number of expected reports	ESU010	Number of sites assessed	4	4	100%	6	6	100%	10	24	42%	10	10	100%	1	1	Presence
Staff with capacity to generate summary reports with eRHIS	National/regional summary for a month	ESU011a_1	Number of sites assessed	4	4	100%	1	6	17%	3	24	13%	5	10	50%	1	1	Presence
	National/regional summary for a quarter	ESU011a_2	Number of sites assessed	4	4	100%	2	6	33%	3	24	13%	6	10	60%	1	1	Presence
	National/regional summary for the year	ESU011a_3	Number of sites assessed	4	4	100%	2	6	33%	3	24	13%	6	10	60%	1	1	Presence
	District summary for a month	ESU011b_1	Number of sites assessed	4	4	100%	6	6	100%	6	24	25%	10	10	100%	0	1	Absence
	District summary for a quarter	ESU011b_2	Number of sites assessed	4	4	100%	6	6	100%	6	24	25%	10	10	100%	0	1	Absence
	District summary for the year	ESU011b_3	Number of sites assessed	4	4	100%	6	6	100%	6	24	25%	10	10	100%	0	1	Absence
	Health facility summary for a month	ESU011c_1	Number of sites assessed	4	4	100%	5	6	83%	23	24	96%	9	10	90%	1	1	Presence
	Health facility summary for a quarter	ESU011c_2	Number of sites assessed	4	4	100%	5	6	83%	22	24	92%	9	10	90%	1	1	Presence
	Health facility summary for the year	ESU011c_3	Number of sites assessed	4	4	100%	5	6	83%	22	24	92%	9	10	90%	1	1	Presence
	Community-level SDP summary for a month	ESU011d_1	Number of sites assessed	2	4	50%	4	6	67%	5	24	21%	6	10	60%	1	1	Presence
	Community-level SDP summary for a quarter	ESU011d_2	Number of sites assessed	2	4	50%	5	6	83%	5	24	21%	7	10	70%	1	1	Presence
	Community-level SDP summary for the year	ESU011d_3	Number of sites assessed	2	4	50%	5	6	83%	5	24	21%	7	10	70%	1	1	Presence
	Aggregated indicator	Sum of numerators	12*Number of sites assessed	42	48	88%	52	72	72%	109	288	38%	94	120	78%			
Ability to calculate coverage indicators																		
Staff able to calculate coverage indicators using eRHIS	Indicator 1 National	ESU012_1a	Number of sites assessed	3	4	75%	0	6	0%	3	24	13%	3	10	30%	1	1	Presence
	Indicator 1 Regional	ESU012_1b	Number of sites assessed	4	4	100%	2	6	33%	3	24	13%	6	10	60%	1	1	Presence
	Indicator 1 District	ESU012_1c	Number of sites assessed	4	4	100%	5	6	83%	4	24	17%	9	10	90%	1	1	Presence
	Indicator 1 Health facility	ESU012_1d	Number of sites assessed	4	4	100%	4	6	67%	14	24	58%	8	10	80%	1	1	Presence
	Indicator 1 Community-level SDP	ESU012_1e	Number of sites assessed	3	4	75%	1	6	17%	1	24	4%	4	10	40%	1	1	Presence
	Indicator 2 National	ESU012_2a	Number of sites assessed	3	4	75%	0	6	0%	3	24	13%	3	10	30%	1	1	Presence
	Indicator 2 Regional	ESU012_2b	Number of sites assessed	4	4	100%	2	6	33%	3	24	13%	6	10	60%	1	1	Presence
	Indicator 2 District	ESU012_2c	Number of sites assessed	4	4	100%	5	6	83%	4	24	17%	9	10	90%	1	1	Presence
	Indicator 2 Health facility	ESU012_2d	Number of sites assessed	4	4	100%	4	6	67%	15	24	63%	8	10	80%	1	1	Presence
	Indicator 2 Community-level SDP	ESU012_2e	Number of sites assessed	3	4	75%	1	6	17%	1	24	4%	4	10	40%	1	1	Presence
	Indicator 3 National	ESU012_3a	Number of sites assessed	2	4	50%	0	6	0%	3	24	13%	2	10	20%	1	1	Presence
	Indicator 3 Regional	ESU012_3b	Number of sites assessed	3	4	75%	2	6	33%	3	24	13%	5	10	50%	1	1	Presence
	Indicator 3 District	ESU012_3c	Number of sites assessed	3	4	75%	5	6	83%	4	24	17%	8	10	80%	1	1	Presence
	Indicator 3 Health facility	ESU012_3d	Number of sites assessed	3	4	75%	4	6	67%	13	24	54%	7	10	54%	1	1	Presence
	Indicator 3 Community-level SDP	ESU012_3e	Number of sites assessed	2	4	50%	1	6	17%	1	24	4%	3	10	70%	1	1	Presence
	Aggregated indicator	Sum of numerators	15*Number of sites assessed	49	60	82%	36	90	40%	75	360	21%	85	150	57%			
Data analysis																		
Staff can generate major causes institution-based newborn mortality	User can generate major causes of institution-based (inpatient, emergency) mortality	ESU015	Number of sites assessed	0	4	0%	0	6	0%	5	24	21%	0	10	0%	0	1	Absence
Staff can generate major newborn morbidity diagnoses	User can generate major morbidity diagnoses for inpatient and outpatient services	ESU016	Number of sites assessed	1	4	25%	1	6	17%	6	24	25%	2	10	20%	0	1	Abscence
Data visualization																		
Staff able to use eRHIS data visualization features to present data	Indicator 1 User can generate time trend graphs	ESU014_1a	Number of sites assessed	4	4	100%	2	6	33%	13	24	54%	6	10	60%	1	1	Presence
	Indicator 1 User can generate bar graphs for comparing facilities, districts, or regions	ESU014_1b	Number of sites assessed	4	4	100%	2	6	33%	11	24	46%	6	10	60%	1	1	Presence
	Indicator 1 User can generate thematic maps, by region, district, or health facility	ESU014_1c	Number of sites assessed	2	4	50%	3	6	50%	9	24	38%	5	10	50%	1	1	Presence
	Indicator 2 User can generate time trend graphs	ESU014_2a	Number of sites assessed	2	4	50%	1	6	17%	11	24	46%	3	10	30%	1	1	Presence
	Indicator 2 User can generate bar graphs for comparing facilities, districts, or regions	ESU014_2b	Number of sites assessed	2	4	50%	1	6	17%	9	24	38%	3	10	30%	1	1	Presence
	Indicator 2 User can generate thematic maps, by region, district, or health facility	ESU014_2c	Number of sites assessed	1	4	25%	2	6	33%	8	24	33%	3	10	30%	1	1	Presence
	Aggregate indicator	Sum of numerators	6*Number of sites assessed	15	24	62%	11	36	31%	61	144	42%	26	60	43%			

eRHIS= electronic Routine Health Information System; MoH = Ministry of Health; SDP = service delivery point.

*definition of the IMPULSE study; it aggregates regional data offices and district data offices.

In green are marked the indicators with a reported percentage between 90% and 100%, in yellow those between 80% and 89% and in red those below 80%.

Table S9. Behavioral factors.

Behavioral factors		PRISM formula		Regional data offices			District data offices			Facilities			Subnational*			MoH		
**Knowledge**	Description indicator	Numerator	Denominator		Denominator	Percentage	Numerator	Denominator	Percentage	Numerator	Denominator	Percentage	Numerator	Denominator	Percentage	Numerator	Denominator	Percentage
Respondent's knowledge on rationale for RHIS data	Knowledge of the rationale for RHIS data	U1a<br>U1b<br>U1c<br>U1d<br>U1e<br>U1f	3*Number of respondents	80	144	56%	79	198	40%	508	1404	36%	159	342	46%	20	36	56%
Respondent's knowledge on data quality checks	Knowledge of data quality checking methods	U2<br>U3	3*Number of respondents	39	48	81%	50	66	76%	238	468	51%	89	114	78%	7	12	58%
**Motivation level among staff**																		
Respondent's perceived motivation to perform RHIS tasks	Staff motivation level to perform RHIS tasks	Inverse BC1<br>Inverse BC2<br>Inverse BC3<br>BC4<br>BC5<br>BC6<br>Inverse BC7	35 * Number of respondents	186	280	66%	274	385	71%	1759	2730	64%	460	665	69%	48	70	69%
**Data quality assurance**																		
Respondents believe they can check data accuracy	Respondent believes that he or she can check data accuracy	SE1	10 x number of respondents	75	80	94%	105	110	95%	629	780	81%	180	190	95%	18	20	90%
**Calculating Indicators**																		
Respondents believe they can calculate rates correctly	Respondent believes that he or she can calculate percentages/rates correctly	SE2	10 x number of respondents	78	80	98%	108	110	98%	611	780	78%	186	190	98%	19	20	95%
**Data Presentation**																		
Respondents believe they can plot a trend on a chart	Respondent believes that he or she can plot a trend on a chart	SE3	10 x number of respondents	75	80	94%	108	110	98%	560	780	72%	183	190	96%	19	20	95%
**Data Interpretation**																		
Respondents believe they can explain the results	Respondent believes that he or she can explain the implication of the results of data analysis	SE4	10 x number of respondents	73	80	91%	96	110	87%	584	780	75%	169	190	89%	19	20	95%
**Use of Information**																		
Respondents believe they can use data for decisions	Respondent believes that he or she can use data for identifying service performance gaps and setting performance targets<br>Respondent believes that he or she can use data for making operational/management decisions	SE5<br>SE6	10 x number of respondents	138	160	86%	183	220	83%	1201	1560	77%	321	380	84%	35	40	88%
**Actual Skills to Perform RHIS Tasks**																		
Respondent's competence in calculating indicators	Competence level in calculating indicators	CD1<br>CD3<br>CD4	3*Number of respondents	17	24	71%	20	33	61%				37	57	65%	5	6	83%
		CF1<br>CF3a<br>CF3b<br>CS3<br>CS4	5*Number of respondents							81	390	21%						
Respondent's competence in plotting data/charts	Competence level in plotting data/preparing charts	CD2a_n	Number of respondents	4	8	50%	3	11	27%				7	19	37%	1	2	50%
		CS2a_n	Number of respondents							21	78	27%						
Respondent's competence in interpreting data	Competence level in interpreting data	CD2b_n<br>CD2c1_n<br>CD2c2_n	4*Number of respondents	25	32	78%	31	44	70%				56	76	74%	7	8	88%
		CF2b_n<br>CF2c1_n<br>CFc2_n<br>CS2b_n<br>CS2c_n	7*Number of respondents							87	546	16%						
Respondent's competence in problem solving	Competence level in problem solving	PSa<br>PSb<br>PSc	10*Number of respondents	51	80	64%	60	110	55%	313	780	40%	111	190	58%	8	20	40%
Respondent's competence in use of information	Competence level in the use of information	CD2d1_n<br>CD2d2_n<br>CD2d3_n	3*Number of respondents	15	24	62%	22	33	67%				37	57	65%	5	6	83%
		CF2d1_n<br>CF2d2_n<br>CS2d1_n<br>CS2d2_n	4*Number of respondents							65	312	21%						

RHIS=Routine Health Information System, MoH = Ministry of Health.

*definition of the IMPULSE study; it aggregates regional data offices and district data offices.

Grey colored portions of the table indicate that the indicator is not recorded for that specific site level; in green are marked the indicators with a reported percentage between 90% and 100%, in yellow those between 80% and 89% and in red those below 80%.

Table S10. Data management.

Data managment		PRISM formula		District data offices		
Evidence of data analysis taking place				Numerator	Denominator	Percentage
Level of data analysis practice	Aggregated/summary RHIS report for newborn and stillbirth data within the past three months.	DQ036a	Number of districts assessed	5	6	83%
	Demographic data on the catchment population of the district for calculating newborn and stillbirth impact indicators	DQ036b	Number of districts assessed	4	6	67%
	Indicators for impact (e.g., neonatal mortality rate, stillbirth rate) calculated for each facility catchment area in the district within the past three months.	DQ036c	Number of districts assessed	3	6	50%
	Comparisons among facilities in the district for impact indicators (e.g., neonatal mortality rate, stillbirth rate).	DQ036d	Number of districts assessed	4	6	67%
	Comparisons with district/national targets for newborn mortality rate and stillbirth rate	DQ036e	Number of districts assessed	5	6	83%
	Comparisons of data over time (monitoring trends) for impact indicators (e.g., neonatal mortality rate, stillbirth rate).	DQ036f	Number of districts assessed	4	6	67%
	Comparisons of sex-disaggregated data (e.g., newborn mortality rate, stillbirth rate, etc.).	DQ036g	Number of districts assessed	1	6	17%
	Comparisons of service coverage (e.g., Kangaroo mother care (KMC) initiation, stillbirth, etc.	DQ036h	Number of districts assessed	4	6	67%
	Aggregated indicator	Sum of numerators	8*Number of districts assessed	30	48	62%
	Aggregated/summary RHIS report for newborn and stillbirth data within the past three months	FQ070a	Number of facilities assessed			
	Demographic data on the catchment population of the health facility for calculating newborn and stillbirth impact indicators	FQ070b	Number of facilities assessed			
	Indicators for impact (e.g., neonatal mortality rate, stillbirth rate, low birthweight rate) calculated for the health facility catchment area within the past three months.	FQ070c	Number of facilities assessed			
	Comparisons between health facility and district/national targets for newborns and stillbirths	FQ070d	Number of facilities assessed			
	Comparisons of data over time, i.e., monitoring trends for impact indicators (e.g., neonatal mortality rate, stillbirth rate, low birthweight rate).	FQ070e	Number of facilities assessed			
	Comparisons of sex-disaggregated data (e.g., for neonatal mortality rate, stillbirth rate, low birthweight rate	FQ070f	Number of facilities assessed			
	Comparisons of service coverage (e.g., early initiation of breastfeeding, Kangaroo mother care (KMC))	FQ070g	Number of facilities assessed			
	Aggregated indicator	Sum of numerators	7*Number of facilities assessed			
Data visualization						
Sites availability of data visuals showing achievements	District office prepares data visuals showing achievements toward targets	DU003	Number of districts assessed	6	6	100%
	Health facility prepares data visuals showing achievements toward targets	FU003	Number of facilities assessed			
Feedback mechanism in place						
Sites with feedback reports sent in the last 3	District sent feedback reports using RHIS information to health facilities in the past three months	DU009	Number of districts assessed	6	6	100%
	Health facility received feedback reports from the district office/Ministry of Health (MOH) based on RHIS information in the past three months	FU009	Number of facilities assessed			
Data quality a	Description indicator	Numerator	Denominator	Numerator	Denominator	Percentage
Sites with data quality control standards in place	District has a designated person to review the quality of compiled newborn and stillbirth data prior to submission to the next level	DQ011	Number of districts assessed	5	6	83%
	District has a written guidelines for data review and quality control	DQ012b	Number of districts assessed	6	6	100%
	Designated staff trained on data review and quality control	DQ013b	Number of districts assessed	3	6	50%
	District has a written guidelines on routine health data quality assessment/assurance	DQ029	Number of districts assessed	6	6	100%
	District conducts data quality assessments for newborn and stillbirth data at health facilities	DQ030	Number of districts assessed	6	6	1000%
	District uses data quality assessments tools,, routine data quality assessment, and in-built electronic data quality validation rules/system	DQ031	Number of districts assessed	6	6	100%
	District mantains a record of health facility data qualit assessments for newborn and stillbirth data conducted in the past 12 months	DQ032	Number of districts assessed	6	6	100%
	Districts maintains a record of feedback to health facilities on data quality assessments for newborn and stillbirthh data findings	DQ033	Number of districts assessed	6	6	100%
	Aggregated indicator	Sum of numerators	8*Number of districts assessed	44	48	92%
	Facility has a designated person to revierw the quality of compiled newborn and stillbirth data prior to submission to the next level	FQ012	Number of facilities assessed			
	Facility has designated staff trained in data quality review or data quality check	FQ013b	Number of facilities assessed			
	Facility has written instructions/guidelines on how to perform a data quality review or data quality check	FQ063	Number of facilities assessed			
	Facility conducts regular data accuracy checks	FQ064	Number of facilities assessed			
	Facility has access to data quality self-assessment tools	FQ065	Number of facilities assessed			
	Facility maintains a record of health facility data accuracy self-assessments conducted in the past three months	FQ066	Number of facilities assessed			
	Faciliy maintains records of feedback to staff on data quality self-assessment findings	FQ067	Number of facilities assessed			
	Aggregated indicator	Sum of numerators	7*Number of facilities assessed			

Grey colored portion of the table indicate that the indicator is not recorded either at district or facility level; in green are marked the indicators with a reported percentage between 90% and 100%, in yellow those between 80% and 89% and in red those below 80%. Data management indicators were collected only at district and facility level,

Table S11. Data quality.

Data quality		PRISM formula		District data offices			Facilities		
Availability		Numerator	Denominator	Numerator	Denominator	Percentage	Numerator	Denominator	Percentage
Numerator									
Stillbirths	Month 1	DQ024a_3a	DQ023_3a	70	219	32%			
	Month 2	DQ024b_3a	DQ023_3b	70	219	32%			
	Month 3	DQ024c_3a	DQ023_3c	66	219	30%			
	All months	Sum numerators	Sum denominators	206	657	31%			
	Month 1	FQ037_1a (1 to 3)	FQ033=1				21	23	91%
	Month 2	FQ037_2a (1 to 3)	FQ033=1				21	23	91%
	Month 3	FQ037_3a (1 to 3)	FQ033=1				21	23	91%
	All months	Sum numerators	Sum denominators				63	69	91%
Institutional neonatal deaths	Month 1	DQ024a_9a	DQ023_9a	10	219	5%			
	Month 2	DQ024b_9a	DQ023_9b	57	219	26%			
	Month 3	DQ024c_9a	DQ023_9c	10	219	5%			
	All months	Sum numerators	Sum denominators	77	657	12%			
	Month 1	FQ059ND_1a=1 (1 to 3)	FQ055ND				14	23	61%
	Month 2	FQ059ND_2a=1 (1 to 3)	FQ055ND				14	23	61%
	Month 3	FQ059ND_3a=1 (1 to 3)	FQ055ND				14	23	61%
	All months	Sum numerators	Sum denominators				42	69	61%
Low birthweight	Month 1	DQ024a_4a	DQ023_4a	52	219	24%			
	Month 2	DQ024b_4a	DQ023_4b	53	219	24%			
	Month 3	DQ024c_4a	DQ023_4c	56	219	26%			
	All months	Sum numerators	Sum denominators	161	657	25%			
	Month 1	FQ045_1a=1 (1 to 3)	FQ041=1				21	23	91%
	Month 2	FQ045_2a=1 (1 to 3)	FQ041=1				21	23	91%
	Month 3	FQ045_3a=1 (1 to 3)	FQ041=1				21	23	91%
	All months	Sum numerators	Sum denominators				63	69	91%
Bag-mask-ventilation	Month 1	DQ024a_6a	DQ023_6a	37	219	17%			
	Month 2	DQ024b_6a	DQ023_6b	41	219	19%			
	Month 3	DQ024c_6a	DQ023_6c	38	219	17%			
	All months	Sum numerators	Sum denominators	116	657	18%			
	Month 1	FQ059_1a=1 (1 to 3)	FQ055=1				20	23	87%
	Month 2	FQ059_2a=1 (1 to 3)	FQ055=1				20	23	87%
	Month 3	FQ059_3a=1 (1 to 3)	FQ055=1				20	23	87%
	All months	Sum numerators	Sum denominators				60	69	87%
Kangaroo mother care	Month 1	DQ024a_8a	DQ023_8a	24	219	11%			
	Month 2	DQ024b_8a	DQ023_8b	28	219	13%			
	Month 3	DQ024c_8a	DQ023_8c	29	219	13%			
	All months	Sum numerators	Sum denominators	81	657	12%			
	Month 1	FQ059KMC_1a=1 (1 to 3)	FQ055KMC=1				21	22	95%
	Month 2	FQ059KMC_2a=1 (1 to 3)	FQ055KMC=1				21	22	95%
	Month 3	FQ0599KMC_3a=1 (1 to 3)	FQ055KMC=1				21	22	95%
	All months	Sum numerators	Sum denominators				63	66	95%
Neonatal sepsis	Month 1	DQ024a_10a	DQ023_10a	100	219	46%			
	Month 2	DQ024b_10a	DQ023_10b	113	219	52%			
	Month 3	DQ024c_10a	DQ023_10c	108	219	49%			
	All months	Sum numerators	Sum denominators	321	657	49%			
	Month 1	FQ059NS_1a=1 (1 to 3)	FQ055NS=1				9	18	50%
	Month 2	FQ059NS_2a=1 (1 to 3)	FQ055NS=1				9	18	50%
	Month 3	FQ059NS_3a=1 (1 to 3)	FQ055NS=1				9	18	50%
	All months	Sum numerators	Sum denominators				27	54	50%
Uterotonics prevent PPH	Month 1	FQ059UT_1a	FQ055UT=1				2	3	67%
	Month 2	FQ059UT_2a	FQ055UT=1				2	3	67%
	Month 3	FQ059UT_3a	FQ055UT=1				2	3	67%
	All months	Sum numerators	Sum denominators				6	9	67%
Denominator									
Total births	Month 1	DQ024a_1a	DQ023_1a	210	219	96%			
	Month 2	DQ024b_1a	DQ023_1b	215	219	98%			
	Month 3	DQ024c_1a	DQ023_1c	211	219	96%			
	All months	Sum numerators	Sum denominators	636	657	97%			
	Month 1	FQ021_1a (1 to 3)	FQ017=1				21	23	91%
	Month 2	FQ021_2a (1 to 3)	FQ017=1				21	23	91%
	Month 3	FQ021_3a (1 to 3)	FQ017=1				21	23	91%
	All months	Sum numerators	Sum denominators				63	69	91%
Live births	Month 1	DQ024a_2a	DQ023_2a	213	219	97%			
	Month 2	DQ024b_2a	DQ023_2b	216	219	99%			
	Month 3	DQ024c_2a	DQ023_2c	213	219	97%			
	All months	Sum numerators	Sum denominators	642	657	98%			
	Month 1	FQ029_1a (1 to 3)	FQ025=1				21	23	91%
	Month 2	FQ029_2a (1 to 3)	FQ025=1				21	23	91%
	Month 3	FQ029_3a (1 to 3)	FQ025=1				21	23	91%
	All months	Sum numerators	Sum denominators				63	69	91%

Completeness 1									
Numerator									
Stillbirths	Month 1	DQ024a_3b	DQ023_3a	70	219	32%			
	Month 2	DQ024b_3b	DQ023_3b	70	219	32%			
	Month 3	DQ024c_3b	DQ023_3c	66	219	30%			
	All months	Sum numerators	Sum denominators	206	657	31%			
	Month 1	FQ037_1a=1	FQ033=1				21	23	91%
	Month 2	FQ037_2a=1	FQ033=1				21	23	91%
	Month 3	FQ037_3a=1	FQ033=1				21	23	91%
	All months	Sum numerators	Sum denominators				63	69	91%
Institutional neonatal deaths	Month 1	DQ024a_9b	DQ023_9a	9	219	4%			
	Month 2	DQ024b_9b	DQ023_9b	57	219	26%			
	Month 3	DQ024c_9b	DQ023_9c	10	219	5%			
	All months	Sum numerators	Sum denominators	77	657	12%			
	Month 1	FQ059ND_1a=1	FQ055ND=1				14	23	61%
	Month 2	FQ059ND_2a=1	FQ055ND=1				14	23	61%
	Month 3	FQ059ND_3a=1	FQ055ND=1				14	23	61%
	All months	Sum numerators	Sum denominators				42	69	61%
Low birthweight	Month 1	DQ024a_4b	DQ023_4a	52	219	24%			
	Month 2	DQ024b_4b	DQ023_4b	53	219	24%			
	Month 3	DQ024c_3b	DQ023_4c	56	219	26%			
	All months	Sum numerators	Sum denominators	161	657	25%			
	Month 1	FQ045_1a=1	FQ041=1				21	23	91%
	Month 2	FQ045_2a=1	FQ041=1				21	23	91%
	Month 3	FQ045_3a=1	FQ041=1				21	23	91%
	All months	Sum numerators	Sum denominators				21	69	91%
Bag-mask-ventilation	Month 1	DQ024a_6b	DQ023_6a	37	219	17%			
	Month 2	DQ024b_6b	DQ023_6b	41	219	19%			
	Month 3	DQ024c_6b	DQ023_6c	38	219	17%			
	All months	Sum numerators	Sum denominators	116	657	18%			
	Month 1	FQ059_1a=1	FQ055=1				20	23	87%
	Month 2	FQ059_2a=1	FQ055=1				20	23	87%
	Month 3	FQ059_3a=1	FQ055=1				20	23	97%
	All months	Sum numerators	Sum denominators				60	69	87%
Kangaroo mother care	Month 1	DQ024a_8b	DQ023_8a	24	219	11%			
	Month 2	DQ024b_8	DQ023_8b	28	219	13%			
	Month 3	DQ024c_8b	DQ023_8c	29	219	13%			
	All months	Sum numerators	Sum denominators	81	657	12%			
	Month 1	FQ059KMC_1a=1	FQ055KMC=1				21	22	95%
	Month 2	FQ059KMC_2a=1	FQ055KMC=1				21	22	95%
	Month 3	FQ059KMC_3a=1	FQ055KMC=1				21	22	95%
	All months	Sum numerators	Sum denominators				63	66	95%
Neonatal sepsis	Month 1	DQ024a_10b	DQ023_10a	100	219	46%			
	Month 2	DQ024b_10b	DQ023_10b	113	219	52%			
	Month 3	DQ024c_10b	DQ023_10c	108	219	49%			
	All months	Sum numerators	Sum denominators	321	657	49%			
	Month 1	FQ059NS_1a=1	FQ055NS=1				9	18	50%
	Month 2	FQ059NS_2a=1	FQ055NS=1				9	18	50%
	Month 3	FQ059NS_3a=1	FQ055NS=1				9	18	50%
	All months	Sum numerators	Sum denominators				27	54	50%
Uterotonics to prevent PPH	Month 1	FQ059UT_1a=1	FQ055UT=1				2	3	67%
	Month 2	FQ059UT_2a=1	FQ055UT=1				2	3	67%
	Month 3	FQ059UT_3a=1	FQ055UT=1				2	3	67%
	All months	Sum numerators	Sum denominators				6	9	67%
Denominator									
Total births	Month 1	DQ024a_1b	DQ023_1a	210	219	96%			
	Month 2	DQ024b_1b	DQ023_1b	215	219	98%			
	Month 3	DQ024c_1b	DQ023_1c	211	219	96%			
	All months	Sum numerators	Sum denominators	636	657	97%			
	Month 1	FQ021_1a=1	FQ017=1				21	23	91%
	Month 2	FQ021_2a=1	FQ017=1				21	23	91%
	Month 3	FQ021_3a=1	FQ017=1				21	23	91%
	All months	Sum numerators	Sum denominators				63	69	91%
Live births	Month 1	DQ024a_2b	DQ023_2a	213	219	97%			
	Month 2	DQ024b_2b	DQ023_2b	216	219	99%			
	Month 3	DQ024c_2b	DQ023_2c	213	219	97%			
	All months	Sum numerators	Sum denominators	642	657	98%			
	Month 1	FQ029_1a=1	FQ025=1				21	23	91%
	Month 2	FQ029_2a=1	FQ025=1				21	23	91%
	Month 3	FQ029_3a=1	FQ025=1				21	23	91%
	All months	Sum numerators	Sum denominators				63	69	91%

Completeness 2							
**Numerator**							
Stillbirths	Month 1	FQ036_1a=1	FQ033=1		20	23	87%
	Month 2	FQ036_2a=1	FQ033=1		20	23	87%
	Month 3	FQ036_3a=1	FQ033=1		20	23	87%
	All months	Sum numerators	Sum denominators		60	69	87%
Institutional neonatal deaths	Month 1	FQ058ND_1a=1	FQ055ND=1		10	23	43%
	Month 2	FQ058ND_2a=1	FQ055ND=1		10	23	43%
	Month 3	FQ058ND_3a=1	FQ055ND=1		10	23	43%
	All months	Sum numerators	Sum denominators		30	69	43%
Low birthweight	Month 1	FQ044_1a=1	FQ041=1		7	23	30%
	Month 2	FQ044_2a=1	FQ041=1		7	23	30%
	Month 3	FQ044_3a=1	FQ041=1		7	23	30%
	All months	Sum numerators	Sum denominators		21	69	30%
Bag-mask-ventilation	Month 1	FQ058_1a=1	FQ055=1		2	23	9%
	Month 2	FQ058_2a=1	FQ055=1		2	23	9%
	Month 3	FQ058_3a=1	FQ055=1		2	23	9%
	All months	Sum numerators	Sum denominators		6	69	9%
Kangaroo mother care	Month 1	FQ058KMC_1a=1	FQ055KMC=1		5	22	23%
	Month 2	FQ058KMC_2a=1	FQ055KMC=1		5	22	23%
	Month 3	FQ058KMC_3a=1	FQ055KMC=1		5	22	23%
	All months	Sum numerators	Sum denominators		15	66	23%
Neonatal sepsis	Month 1	FQ058NS_1a=1	FQ055NS=1		5	6	28%
	Month 2	FQ058NS_2a=1	FQ055NS=1		5	6	28%
	Month 3	FQ058NS_3a=1	FQ055NS=1		5	6	28%
	All months	Sum numerators	Sum denominators		15	18	28%
Uterotonics to prevent PPH	Month 1	FQ058UT_1a=1	FQ055UT=1		1	3	33%
	Month 2	FQ058UT_2a=1	FQ055UT=1		1	3	33%
	Month 3	FQ058UT_3a=1	FQ055UT=1		1	3	33%
	All months	Sum numerators	Sum denominators		3	9	33%
**Denominator**							
Total births	Month 1	FQ020_1a=1	FQ017=1		20	23	87%
	Month 2	FQ020_2a=1	FQ017=1		20	23	87%
	Month 3	FQ020_3a=1	FQ017=1		20	23	87%
	All months	Sum numerators	Sum denominators		60	69	87%
Live births	Month 1	FQ028_1a=1	FQ025=1		18	23	78%
	Month 2	FQ028_2a=1	FQ025=1		18	23	78%
	Month 3	FQ028_3a=1	FQ025=1		18	23	78%
	All months	Sum numerators	Sum denominators		54	69	78%

Accuracy 1						
**Tollerance 0%**						
**Numerator**						
Stillbirths	VF of 1	Number of sites providing the service		4	23	17%
Institutional neonatal deaths	VF of 1	Number of sites providing the service		4	23	17%
Low birth weight	VF of 1	Number of sites providing the service		3	23	13%
Bag mask ventilation	VF of 1	Number of sites providing the service		6	23	26%
Kangaroo mother care	VF of 1	Number of sites providing the service		6	22	27%
Neonatal sepsis	VF of 1	Number of sites providing the service		2	18	11%
Uterotonics prevent PPH	VF of 1	Number of sites providing the service		2	3	67%
**Denominator**						
Total births	VF of 1	Number of sites providing the service		3	23	13%
Live births	VF of 1	Number of sites providing the service		3	23	13%

Tollerance 20%									
Numerator									
Stillbirths		VF between 0.9 and 1.1	Number of sites providing the service				8	23	35%
Institutional neonatal deaths		VF between 0.9 and 1.1	Number of sites providing the service				14	23	22%
Low birth weight		VF between 0.9 and 1.1	Number of sites providing the service				6	23	26%
Bag mask ventilation		VF between 0.9 and 1.1	Number of sites providing the service				14	23	26%
Kangaroo mother care		VF between 0.9 and 1.1	Number of sites providing the service				7	22	32%
Neonatal sepsis		VF between 0.9 and 1.1	Number of sites providing the service				3	18	17%
Uterotonics prevent PPH		VF between 0.9 and 1.1	Number of sites providing the service				2	3	67%
Denominator									
Total births		VF between 0.9 and 1.1	Number of sites providing the service				14	23	61%
Live births		VF between 0.9 and 1.1	Number of sites providing the service				14	23	61%
Timeliness									
Timeliness of facility reporting	Month 1	DQ020 (1a to 4a)	DQ015	89	219	41%			
	Month 2	DQ020 (1b to 4b)	DQ015	70	219	32%			
	Month 3	DQ020 (1c to 4c)	DQ015	101	219	46%			

PPH = post partum haemorrhage, VF = verification factor.

Grey coloured portion of the table indicate that the indicator is not recorded either at district or facility level; in green are marked the indicators with a reported percentage between 90% and 100%, in yellow those between 80% and 89% and in red those below 80%; completeness 1 is specific to monthly register; completeness 2 is specific to source documents; the calculation of the VF is the ratio between the data reported in the source document (DQ026_1a to 10a, DQ027_2a to 10a and DQ028_1a to 10a) and the data reported in the electronic or paper based report submitted by the district (DQ026_1b to 10bb, DQ027_2bb to 10b and DQ028_1b to 10b). Data quality indicators were collected only at district data office and facility level.

Table S12. Data use.

Use of information		PRISM formula		District data office			Facility		
Use of data to	Description indicator	Numerator	Denominator	Numerator	Denominator	Percentage	Numerator	Denominator	Percentage
Sites produce any report or bulletin	District office	DU006	Number of districts	6	6	100%			
	Health facility	FU006	Number of facilities				16	24	67%
Use of									
Sites with use of RHIS data for performance monitoring (all sites)	Discussion RHIS	DU016a	5*Number of districts assessed	9	30	30%			
	Decisions based on	DU016b							
	Follow up action	DU016c							
	Key performance	DU016d_1 to							
	Decisions made	DU017_1 to							
	Discussion RHIS	FU016a	5*Number of facilities assessed				23	120	19%
	Decisions based on	FU016b							
	Follow up action	FU016c							
	Key performance	FU016d							
	Decisions made	FU017							
Sites with use of RHIS data for performance monitoring (meeting minutes)	Discussion RHIS	DU016a	5*Number of districts assessed (with meeting minutes)	9	10	90%			
	Decisions based on	DU016b							
	Follow up action	DU016c							
	Key performance	DU016d_1 to							
	Decisions made	DU017_1 to							
	Discussion RHIS	FU016a	5*Number of facilities assessed (with meeting minutes)				23	35	66%
	Decisions based on	FU016b							
	Follow up action	FU016c							
	Key performance	FU016d							
	Decisions made	FU017							
Sites with key performance targets discussed	Coverage of service	DU016d_1	7*Number of districts assessed	9	42	21%			
	Hospital/health cente	DU016d_2							
	Disease data (e.g., top	DU016d_3							
	Identification of emer	DU016d_4							
	Medicine stockouts	DU016d_5							
	Human resource (HR)	DU016d_6							
	Sex-disaggregated da	DU016d_7							
	Coverage of service li	FU016d_1	7*Number of facilities assessed				28	168	17%
	Hospital/health cente	FU016d_2							
	Disease data (e.g., top	FU016d_3							
	Identification of emer	FU016d_4							
	Commodity stockout	FU016d_5							
	HR management	FU016d_6							
	Sex-disaggregated da	FU016d_7							
Sites with decisions made based on the performance discussions	Formulation of plans	DU017_1	11*Number of districts assessed	17	66	26%			
	Budget preparation	DU017_2							
	Budget reallocation	DU017_3							
	Medicine supply and c	DU017_4							
	HR management (trai	DU017_5							
	Advocacy for policy, p	DU017_6							
	Health services (preve	DU017_7							
	Promotion of service c	DU017_8							
	Reducing the gender g	DU017_9							
	Involvement of the co	DU017_10							
	No action required at	DU017_11							
	Formulation of plans	FU017_1	9*Number of facilities assessed				26	216	12%
	Budget preparation	FU017_2							
	Budget reallocation	FU017_3							
	Medicine supply and c	FU017_4							
	HR management (trai	FU017_5							
	Advocacy for policy, p	FU017_6							
	Promotion of service c	FU017_7							
	Reducing the gender g	FU017_8							
	No action required at	FU017_9							
Sites with issues covered in annual plans demonstrating RHIS data use	Service coverage	DU022_1	7*DU020=1	28	42	67%			
	Health facility perforr	DU022_2							
	Diseases	DU022_3							
	Emerging issues/epide	DU022_4							
	Medicine stockouts	DU022_5							
	HR management	DU022_6							
	Gender disparity in he	DU022_7							
	Service coverage	FU021_1	7*FU019=1				94	147	64%
	Health facility perforr	FU021_2							
	Diseases	FU021_3							
	Emerging issues/epide	FU021_4							
	Commodity stockouts	FU021_5							
	HR management	FU021_6							
	Gender disparity in he	FU021_7							
Data									
Sites required to disseminate	District has to	DU023	Number of districts	5	6	83%			
	Health facility has to s	FU028	Number of facilities				11	24	46%
Sites using/sharing data from the health indicators performance report	Reports/presentation	DU025	3*DU023=1	10	15	67%			
	Website is updated	DU026							
	District performance	DU027							
	Reports/presentation	FU030	3*FU028=1				32	33	97%
	Website is updated	FU031							
	District performance	FU032							

RHIS = routine health information system.

Grey colored portion of the table indicate that the indicator is not recorded either at district or facility level; in green are marked the indicators with a reported percentage between 90% and 100%, in yellow those between 80% and 89% and in red those below 80%. Data use indicators were collected only at district data office and facility level.

Table S13. Number of indicators in each major category by facility type.

Major category	Subnational data offices		Healthcare facilities		Overall	
	N	%	N	%	N	%
	N = 183		N = 195		N = 378	
Organizational factors	57	31.1	47	24.1	104	27.5
Technical factors	36	19.7	36	18.5	72	19
Behavioral factors	13	7.1	13	6.7	26	6.9
Data management	18	9.8	16	8.2	34	9
Data quality	19	10.4	45	23.1	64	16.9
Use of information	40	21.9	38	19.4	78	20.6

Major categories refer to the domains defined by PRSIM framework [25], as displayed in **Figure 3.**

FIGURES

Figure S1. PRISM Framework and conceptual model.

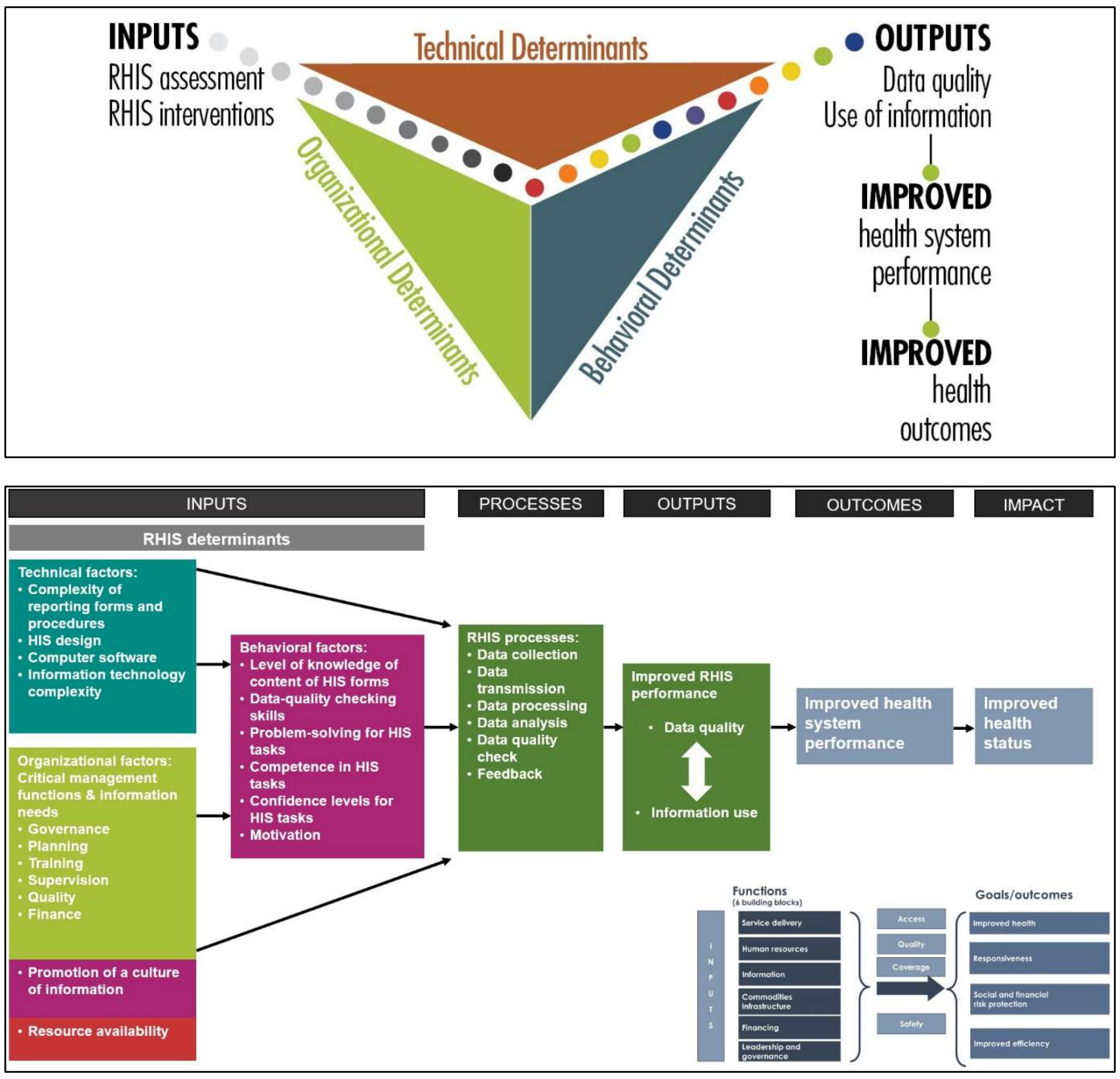

Aqil A, Lippeveld T, Hozumi D. PRISM framework: a paradigm shift for designing, strengthening and evaluating routine health information systems. Health Policy Plan. 2009;24:217–28. doi:10.1093/heapol/czp010.

HIS = health information system, PRISM = performance of routine information system management, RHIS = routine health information system.

Figure S2. IMPULSE Study End users' perspectives on the need for improvement in RHIS (252 respondents).

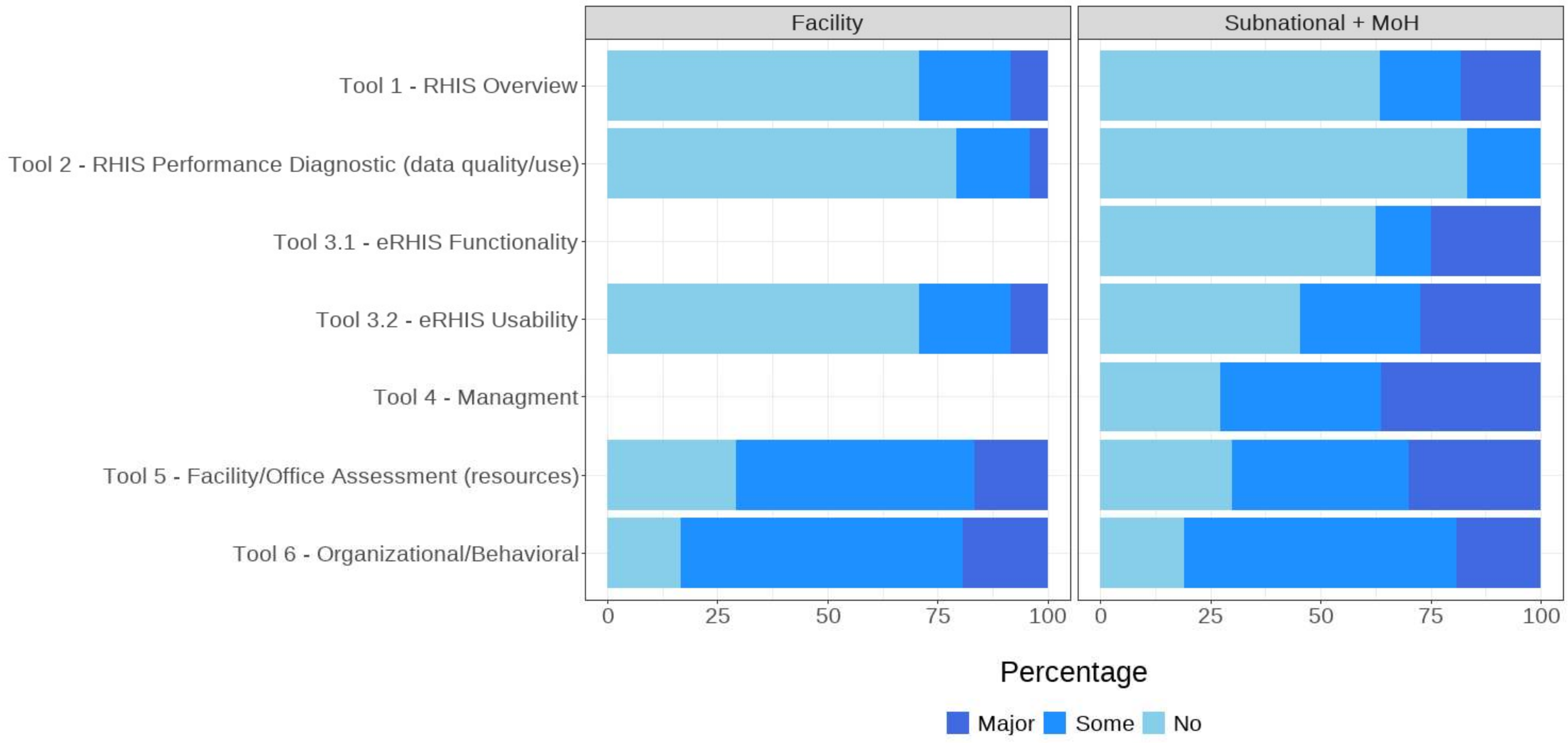

eRHIS = electronic Routine Health Information System, MoH = Ministry of Health.

Figure S3. Gap between respondents' competence and confidence in carrying out RHIS tasks.

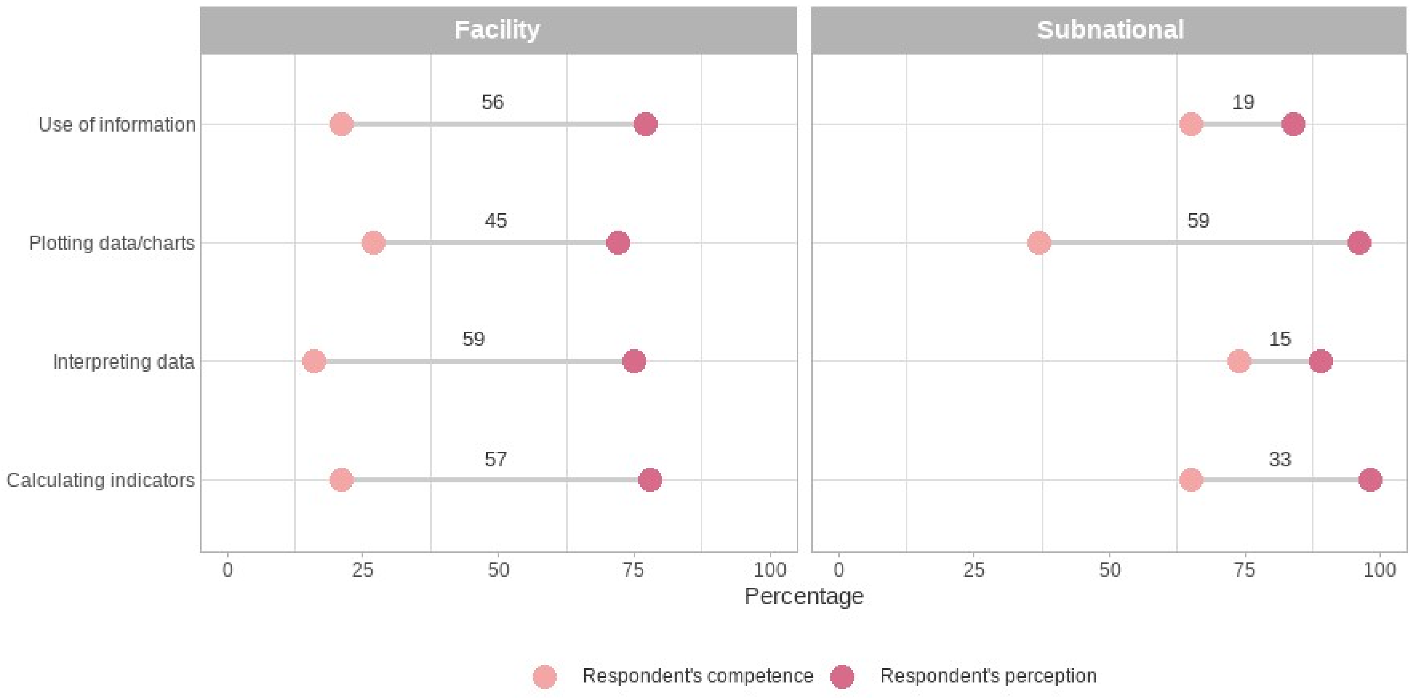

RHIS = routine health information system.
The number above the line indicates the percentage points difference.
